# An algorithm to classify homologous series within compound datasets

**DOI:** 10.1186/s13321-022-00663-y

**Published:** 2022-12-13

**Authors:** Adelene Lai, Jonas Schaub, Christoph Steinbeck, Emma L. Schymanski

**Affiliations:** 1 Luxembourg Centre for Systems Biomedicine (LCSB), University of Luxembourg, 6 Avenue du Swing, 4367 Belvaux, Luxembourg; 2 Institute for Inorganic and Analytical Chemistry, Friedrich Schiller University Jena, Lessing Strasse 8, 07743 Jena, Germany

**Keywords:** RDKit, Fragmentation, Algorithm, Scaffolds, Homologous series, Polymers, Environmental chemistry, Natural products, Exposomics, Pattern recognition

## Abstract

Homologous series are groups of related compounds that share the same core structure attached to a motif that repeats to different degrees. Compounds forming homologous series are of interest in multiple domains, including natural products, environmental chemistry, and drug design. However, many homologous compounds remain unannotated as such in compound datasets, which poses obstacles to understanding chemical diversity and their analytical identification via database matching. To overcome these challenges, an algorithm to detect homologous series within compound datasets was developed and implemented using the RDKit. The algorithm takes a list of molecules as SMILES strings and a monomer (i.e., repeating unit) encoded as SMARTS as its main inputs. In an iterative process, substructure matching of repeating units, molecule fragmentation, and core detection lead to homologous series classification through grouping of identical cores. Three open compound datasets from environmental chemistry (NORMAN Suspect List Exchange, NORMAN-SLE), exposomics (PubChemLite for Exposomics), and natural products (the COlleCtion of Open NatUral producTs, COCONUT) were subject to homologous series classification using the algorithm. Over 2000, 12,000, and 5000 series with CH_2_ repeating units were classified in the NORMAN-SLE, PubChemLite, and COCONUT respectively. Validation of classified series was performed using published homologous series and structure categories, including a comparison with a similar existing method for categorising PFAS compounds. The OngLai algorithm and its implementation for classifying homologues are openly available at: https://github.com/adelenelai/onglai-classify-homologues.

**Supplementary Information:**

The online version contains supplementary material available at 10.1186/s13321-022-00663-y.

## Introduction

**Fig. 1:**
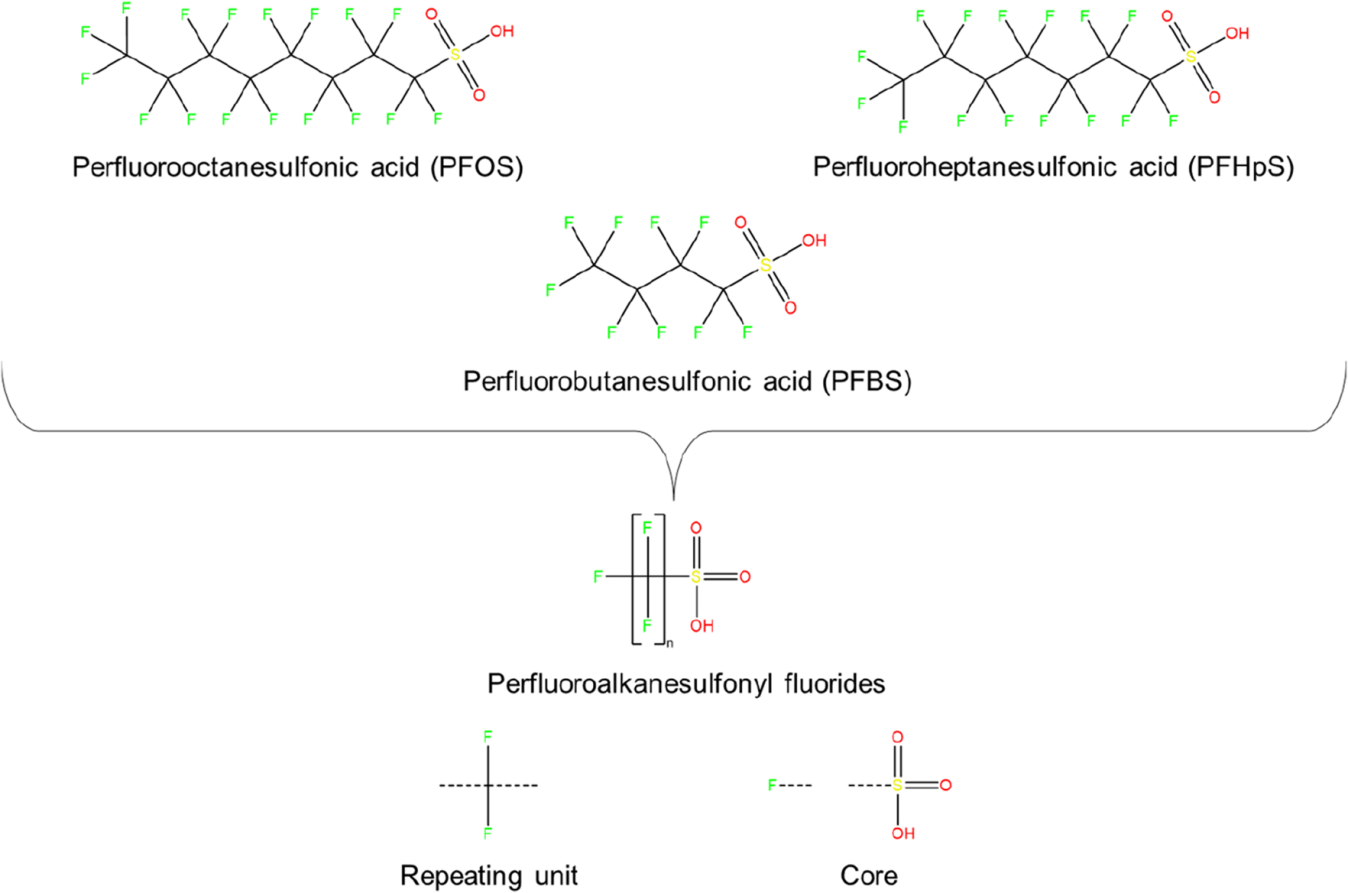
Example of a PFAS homologous series with general formula C_n_F_2n+1_SO_3_H. The series can be expressed using a generic structure that comprises a repeating unit (CF_2_) and core(s), which may be one intact fragment or multiple disconnected fragments, as is the case here

Homologous series are groups of compounds that share the same core structure with varying attached repeating chemical subunits. These structurally-related compounds occur in many areas of chemistry and can be represented by Markush structures [1], as in the patent literature, or as general molecular formulae, for example C_n_F_2n+1_SO_3_H (Fig. 1). In drug design, homologation is used as a molecular modification strategy to construct series for lead optimisation [2], while homologous series are prominent in pesticide synthesis [3], food [4], and material science [5], as well as formulation chemistry [6] for applications in myriad products such as cosmetics, surfactants, and pharmaceuticals. In nature, homologous series occur as natural products of multiple organisms including bacteria [7], fungi [8], marine sponges [9, 10], birds [11], bees [12], and avocados [13]. In the environment, synthetic compounds consisting of homologous series are considered anthropogenic pollutants, for example, surfactants that have been identified extensively in wastewater [14–17], and are classified as High Production Volume chemicals because of their widespread production and use. Other classes of environmental chemical pollutants containing homologous series include the ‘forever chemicals’ i.e., per- and polyfluoroalkyl substances (PFAS) [18–21], as well as technical mixes of polymers such as chlorinated paraffins [22, 23], both of which have been identified extensively in the environment [24, 25], and can be considered as substances of Unknown or Variable composition, Complex reaction products, or Biological materials (UVCBs) [26].

Within compound datasets, having molecules grouped into homologous series can potentially advance several areas of chemistry, for example their analytical identification using liquid chromatography-high resolution mass spectrometry (LC-HRMS). As the structural similarity of homologous compounds can result in a trend in physicochemical properties, homologous series often exhibit characteristic comb-like elution patterns and constant *m/z-*retention time shifts in LC-HRMS data. Such signals are frequently detected in environmental samples, where the constant *m/z* difference between signals is indicative of the repeating unit’s mass and, in some cases, identity. Consequently, their identification is of high interest, especially since they form a relatively significant proportion of environmental unknowns [27] (also known as ‘non-target compounds’). Various data-mining routines [28–30] and screening tools [31] have been developed to address this challenge, which usually involves trying to match spectral features with database entries by mass. However, interpreting the matches to find chemically related identifications i.e., homologous chemical series, remains extremely laborious for two reasons: (1) the sheer number of possible (interconnected) homologues in complex environmental samples, and (2) individual homologous compounds are not linked to each other within databases. Therefore, to address the latter, having homologous compounds classified into series within chemical databases would support environmental chemists in assigning related chemical structure identifications to unknown but likely homologous mass spectral features, series-by-series, where possible. Notably, this advantage extends to chemists seeking to discover novel natural products; if structures of the same homologous series within a combined structural and spectral database are annotated as such, their characteristic spectral similarities and trends can be identified, which could expedite the elucidation of previously unreported members of a given series and hence aid the dereplication of spectral data.

Another area of chemistry that would benefit from classified homologous series in datasets is property prediction. As homologous compounds are structurally similar, structure–property relationships are typically predictable within a given series such that compounds usually share similar properties or show a trend [32], e.g., the Wiener index to predict the boiling points of alkanes [33], Kováts retention indices in gas chromatography to predict analyte retention relative to alkanes [34], or the effect of varying repeating unit chain length on insecticidal activity [13]. In this way, data gaps in physicochemical properties for homologous compounds can be filled using models based on series members that have property data.

Studies of chemical diversity/activity within a given chemical space may also benefit from homologous series classification; instead of focusing on homologous compounds that share repetitive structures and thus similar properties, focus can instead be refined on areas with interesting and varied properties. In other words, grouping together homologous compounds helps eliminate redundancy in the investigated chemical space, as related compounds can be considered group-wise instead of individually. This capability is likely pertinent to medicinal chemists interested in interrogating chemical spaces for diverse properties, or when developing screening decks [35]. In turn, concise representations of a particular chemical space or screening deck may be desired, which could be achieved by general formulae or Markush structures for homologous compounds.

Despite these potential advantages, most compound datasets do not contain homologous compounds classified into series. Instead, homologous compounds typically exist in databases as individual entities without explicit association to one another. To the human eye, homologous series are easily recognisable because of their structural similarity; especially when dealing with simple series and small numbers of chemical structures (10 s to 100 s), a trained chemist can easily classify homologous series by hand as it is a relatively simple, albeit time-consuming pattern recognition task. However, the sizes of today’s compound databases regularly exceed hundreds of structures: as of August 2022, PubChem [36, 37] and ChemSpider [38, 39] contain over 110 million compounds each, while virtual screening libraries used for drug discovery are in the order of billions [40]. Such scale renders manual classification of homologous series impractical. Thus, automated methods using cheminformatic algorithms are needed.

The starting point for automated homologous series classification is the detection of appropriate cores i.e., the common fragment(s) shared by each member of a homologous series. As a series is defined by its core(s), correct core detection by cheminformatic means is as critical as it is challenging. Existing approaches for molecular substructure analysis, in this case to automatically detect cores suitable for homologous series classification, fall into three main categories. The first and most instinctive approach is to consider potential cores as Maximum Common Substructures (MCS) [41, 42]. However, trying to find multiple possible MCS de novo amongst large sets of molecules (> 10,000) is computationally expensive and would likely require additional clustering post-processing steps to obtain the final homologous series. For this purpose, previous work such as Kruger et al.'s clustering approach for chemical series classification [43] has limited applicability because it would not generate core structures specific enough to determine homologous series correctly. An alternative related approach is to exploit pattern-mining algorithms, as homologous series classification can be considered as a task of frequent subgraph mining or graph-based substructure pattern mining [44]. However, these methods require a priori knowledge of a so-called minimum support value, defined as the percentage of all graphs in which a given subgraph must occur. In other words, users must know and specify as input how many series there should be within a given molecule collection, which is impossible to know upfront for most compound datasets. Alternatively, cores could be derived via graph representations of molecules leading to the generation of molecular frameworks as introduced by Bemis and Murcko [45]. However, a significant caveat therein is the required presence of ring systems, which cannot always be assumed.

To address this gap in automated homologous series classification, a free and open algorithm to detect homologous series within compound datasets was developed, which to the best of our knowledge, is the first of its kind. The algorithm was implemented in the RDKit as a Python package called OngLai (pronounced ‘ong-lye’), and is openly and freely available on GitHub [46] (https://github.com/adelenelai/onglai-classify-homologues). (OngLai has a double meaning in Hokkien: literally, pineapple and figuratively, ‘fortune is coming’.) The algorithm input includes a user-specified repeating unit, which forms the basis for the detection of cores that define series. The core fragments are detected without a priori knowledge of their structure, nor how many are present within a given dataset. This result is achieved through successive repeating unit substructure matching and molecule fragmentation steps. Identified homologous series are generated as output, with each compound assigned a number indicating series membership. For a given run of the algorithm, series membership is unique for each molecule as there is only one core fragment result possible once all repeating units have been removed. However, a molecule could in theory belong to multiple homologous series if multiple runs of the algorithm are performed with different settings specified each time, e.g., different repeating unit.

OngLai was used to classify homologous series within three major chemical collections containing compounds from environmental chemistry, exposomics, and natural products. These collections were chosen to highlight the prevalence of homologous compounds in such varied research domains as well as to demonstrate the broad applicability of OngLai. The first of these three collections, the NORMAN Suspect List Exchange (NORMAN-SLE) [47], comprises synthetic chemicals suspected to be present in the environment such as pesticides, pharmaceuticals, surfactants, food-contact chemicals, and those used in industrial applications, like PFAS [48]. The NORMAN-SLE contains 99 so-called ‘suspect’ lists of chemicals hosted by the NORMAN Network, which are used for suspect screening mass spectrometry data generated from measuring environmental samples [47]. The second collection, PubChemLite for Exposomics (PubChemLite), is a subset of PubChem that aims to capture the chemical space relevant for exposomics [49], the study of exposures to chemicals over time. PubChemLite therefore contains chemicals associated with both metabolism and disease (e.g., ‘Biomolecular Interactions and Pathways’, ‘Associated Disorders and Diseases’ etc.), and environmental chemicals (e.g., ‘Agrochemicals’, ‘Drug and Medication Information’ etc*.*). Finally, the COlleCtion of Open Natural prodUcTs (COCONUT) is a compilation of natural product compounds from over 50 open data resources and manually curated datasets from the literature [50, 51]. It is currently the largest open collection of natural products that is freely available online. Natural products consist of compounds produced by organisms such as bacteria, fungi, animals, and plants over the course of various life processes, and because of their potentially high bioactivity, natural products are of great interest for drug discovery. Selected homologous series classified by OngLai in these three collections are reported here.

Additionally, OngLai’s results were validated against published homologous series and PFAS structure categories from the 2018 OECD PFAS definition [52]. The latter is of particular interest to regulatory stakeholders, as PFAS categorisation remains a high-priority task in effort to catalogue and assess the environmental risks of these compounds. A comparison of OngLai to splitPFAS [53], an automated method based on SMARTS [54] matching developed to support PFAS categorisation efforts, was also performed. Previously, PFAS had been manually classified by experts for the 2018 OECD definition to provide common terminology for stakeholders to communicate, research, and regulate these compounds given their widespread uses and potential adverse environmental and health effects. With an ever-growing number of PFAS compound registrations and detections in environmental samples, these so-called ‘forever chemicals’ and their categorisation remain of high priority to various stakeholders interested in their future registration, use, and regulation.

## Methods

### Algorithm and implementation

OngLai was developed and implemented using the RDKit (RDKit version 2021.09.4 [55, 56] and Python version 3.7 [57]) and is openly and freely available on GitHub (https://github.com/adelenelai/onglai-classify-homologues). OngLai is designed to be run in the command line; more information is available in the GitHub README file.

**Fig. 2:**
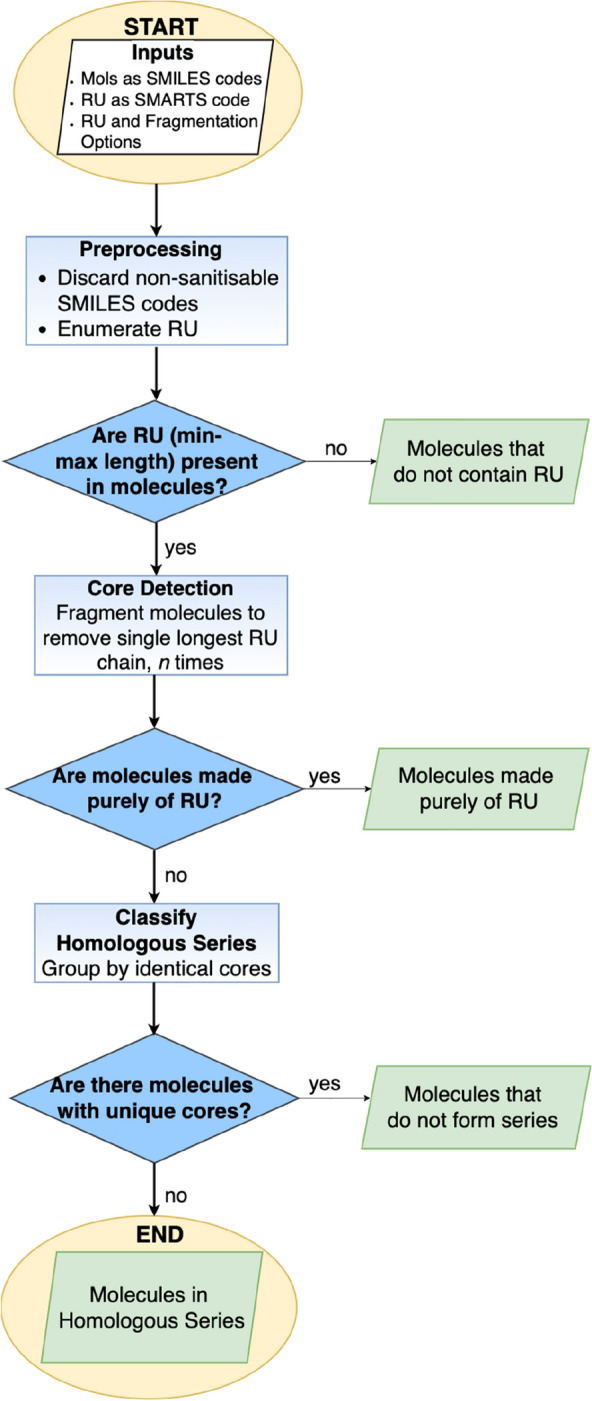
Overview of OngLai algorithm steps to classify homologous series in a set of input molecules. RU represents ‘repeating unit(s)’. Green boxes indicate outputs in SMILES format

Within a set of input molecules given as SMILES strings, OngLai detects homologous series by first detecting cores. It does this by substructure matching chains of user-specified repeating units, then fragmenting the molecules a specified number of times to remove these chains. Molecules with the same remaining core fragments are then grouped together into what is considered a homologous series. The sequence of the algorithm’s steps is provided in Fig. 2 and described in more detail below.

OngLai requires two main inputs: the first is a CSV file with a minimum of two columns containing SMILES representations and molecule names (column names can be specified in the command line according to the dataset used; additional columns will be ignored). In a pre-processing step (Fig. 2), the SMILES codes are parsed and checked for validity i.e., whether they can be converted into sanitised molecule objects within the RDKit. Unparseable SMILES strings are discarded. Molecular sanitisation is a RDKit concept that ensures molecules are ‘reasonable’ i.e., can be represented by Lewis structures with complete octets, and that properties such as ring membership and hybridisation can be calculated for each atom [58].

The second input is a repeating unit of choice, expressed as a SMARTS string. For example, the repeating unit of a series of homologous molecules defined by a growing alkyl chain would be –CH2–, represented as ‘[#6&H2]’ in SMARTS. The definition of a suitable repeating unit is crucial because it determines which cores, and therefore which homologous series, will be detected. Importantly, the starting and terminal atoms of this repeating unit SMARTS string should have open valences such that it is chemically feasible to create a linear chain by concatenating the SMARTS (Fig. 2, ‘Preprocessing’). Thus, the repeating unit SMARTS strings must be defined from connection point to connection point. Example repeating unit SMARTS inputs are provided in Table 1.

**Table 1:** Example repeating units and their SMARTS representations that are suitable for input to OngLai. The default repeating unit is alkyl (CH_2_)

Repeating unit pseudo-SMILES	Repeating unit chemical name	SMARTS (OngLai input)
CH_2_	Alkyl	[#6&H2]
CH_2_CH_2_O	Ethoxy	[#8]-[#6&H2]-[#6&H2]
CH_2_CH_2_CH_2_O	Propoxy	[#8]-[#6&H2]-[#6&H2]-[#6&H2]
CF_2_	Perfluoroalkyl	[#6](-[#9])(-[#9])
CF_2_O	Perfluorinated methyl ether	[#8]-[#6](-[#9])(-[#9])
CF_2_CF_2_O	Perfluorinated ethyl ether	[#8]-[#6](-[#9])(-[#9])-[#6](-[#9])(-[#9])
CH_2_C(CH_3_) = CCH_2_	Isoprene	[#6&H2]-[#6](-[#6&H3]) = [#6]-[#6&H2]

Once pre-processing is complete, repeating unit chains are enumerated according to the first of two user-customisable settings: the minimum and maximum lengths of the repeating unit chains (Table 2). This setting ultimately determines whether repeating units are considered present or absent in the input molecules (Fig. 2, first dark blue rhombus); the default minimum length of 3 is recommended to avoid detections of trivially short repeating unit chains that likely occur frequently in many molecules. Each of the enumerated repeating unit chains is searched within each molecule as potential substructure matches. The result (*HasSubstructMatch* = 1 or 0) is recorded as an element within a NumPy array, one array per input molecule. If the sum of the array elements is equal to zero, the molecule does not contain at least 1 repeating unit chain of the specified minimum length and is then eliminated from further analyses (Fig. 2, first green box). Having established that the remaining molecules contain repeating units, OngLai proceeds with core detection via molecule fragmentation to separate repeating unit chains from core structures. The default setting for the number of molecule fragmentation steps is 2 (Table 2) but can be customised if more than two repeating unit chains are expected to be present in the input molecules. The accuracy of core detection and homologous series classification would technically be unaffected by setting a higher number of fragmentation steps than is actually needed, albeit at the expense of longer computation times. Each time during fragmentation, only one—the longest—repeating unit chain is detected, then removed to ensure ‘clean’ core detection without leftover repeating unit fragments. Importantly, only one repeating unit chain is removed per fragmentation step, even in the case of symmetrical molecules or molecules that otherwise have multiple identical longest repeating unit matches (see Fig. 8 in “Discussion” for further details).

**Table 2:** User-customisable settings of OngLai to specify ‘repeating unit options’ in the command line

Setting	Format	Default
Minimum and maximum lengths of repeating unit chains	Integer	Min. = 3 Max. = 30
No. fragmentation steps	Integer	2

Molecule fragmentation is achieved using RDKit’s *ReplaceCore* function, which introduces a dummy atom at each fragmentation site that is then replaced with a hydrogen atom. However, if the remaining molecule object for a given molecule is empty, it means the input molecule is made purely of repeating units and is reported as such (Fig. 2, second green box). Otherwise, the remaining fragment(s) is considered the core, which can consist of a single or multiple disconnected fragments.

In a final step, molecules are classified into homologous series; those with identical cores (same number and identity of fragments) are deemed members of the same series. Molecules with unique cores, i.e., cores that occur only once in the entire dataset, are considered ‘molecules that do not form series’ (Fig. 2, third green box). In this way, the results of the OngLai are entirely dataset-dependent, as input molecules and consequently their resulting cores are necessarily compared to each other in the homologous series detection process, meaning the co-presence or absence of possible series members determines series classification. A comparison of cores for equality is performed using sanitised canonical RDKit SMILES representations.

A CSV file is generated as output containing the following columns: ‘SMILES’ (only those sanitisable by RDKit), ‘Name’, and ‘series_no’. Series membership is encoded in the ‘series_no’ field, as are the other aforementioned results (Fig. 2, green boxes) as shown in Table 3. Additionally, an overview of the classification results is provided as output, written to a TXT file called ‘classification-results’.

**Table 3:** Interpretation of ‘series_no’ encoding as part of the output from homologous series detection. N+1 is the number of homologous series that were detected by OngLai in a given dataset

Series_no	Interpretation
0–*N*	Molecules that form homologous series
–1	Molecules with no repeating units matches of minimum chain length specified
–2	Molecules made purely of repeating units
–3	Molecules that have repeating units matches of minimum chain length specified but that do not form series (unique cores)

### Datasets

OngLai was applied to three different datasets, NORMAN-SLE [47, 48] used in environmental chemistry, PubChemLite [49, 59] used in exposomics/metabolomics, and COCONUT [50, 51] in natural products research, respectively. All the datasets are openly available (see Additional file 1 Sect. 1.2, Declarations and References).

The NORMAN-SLE dataset used here is an aggregation of the suspect lists that were compiled by the NORMAN Network from various environmental chemistry researchers around the world. The exact dataset originated from the ‘NORMAN Suspect List Exchange Classification’ on PubChem’s Classification Browser (downloaded 2022-03-21) [60, 61]. Using the PubChem Identifier Exchange Service [62], the molecules in NORMAN-SLE were mapped to their Parent CIDs (Operator Type: ‘Parent CID’) to remove salts, charged ions, and mixtures. Stereochemical information is preserved in this process if originally present. Conversion of 115,115 input compounds to Parent CIDs resulted in a final dataset of 98,116 ‘parent’ compounds that were downloaded in CSV format via PubChem. The second dataset, PubChemLite for Exposomics (v.1.8.0), contains 392,465 molecules and was downloaded from Zenodo [49, 59] and used as-is. PubChemLite compounds have both neutral (InChIKey second and third blocks: UHFFFAOYSA-N) and non-neutral stereochemistry. During PubChemLite development, the stereochemical-neutral version was preferentially selected if available, otherwise a structure with stereochemistry was included; further details can be found in the original paper [49]. COCONUT, containing 407,270 molecules (v.11/2021 [50, 51]), was downloaded as SMILES (CDK Unique SMILES [63], i.e., representations without stereochemical information) and used as-is. The specific versions of these datasets used are archived on Zenodo [64]. Specific instructions for running the algorithm on these datasets are available in the GitHub README file https://github.com/adelenelai/onglai-classify-homologues.

### Validation and comparison with existing methods

Validation of OngLai was performed in two ways, by comparing the homologous series it classified in NORMAN-SLE with (1) published homologous series, and (2) published structure categories.

Published homologous series are available in two suspect lists from the NORMAN Suspect List Exchange: *S7 EAWAGSURF *[65], and *S23 EIUBASURF *[66], which both contain surfactant compounds with CH_2_ and CCO repeating units. Homologous series in these two compound lists are explicitly indicated by ‘SurfactantCode’ or ‘Name’ column entries, where members of a given series follow a sequential naming convention e.g., ‘C10-LAS’, ‘C11-LAS’, and ‘C12-LAS’ forming the ‘Cx-LAS’ series, or ‘Amines, coco 10 EO’, ‘Amines, coco 11 EO’, and ‘Amines, coco 12 EO’ forming the ‘Amines, coco x EO’ series (x = 10–12 in both examples). Validation was performed by comparing homologous series classified by OngLai in the NORMAN-SLE dataset with those published in these lists that were downloaded and used as-is.

Published ‘Structure Categories’ determined by experts for the 2018 OECD definition pertain to PFAS compounds containing CF_2_ repeating units obtained from the NORMAN-SLE Classification Tree in PubChem under *S25 OECDPFAS* [52]. These lists of compounds were downloaded from PubChem per structure category via the Identifier Exchange Service and mapped to Parent CID as described above. Validation using these ‘Structure Categories’ proceeded as follows: molecules in a given homologous series classified by OngLai were inspected to see how many structure categories they belonged to, assuming that correctly classified series should have molecules belonging to the same single structure category.

To facilitate validation, a Python script was used to merge OngLai’s CSV output (by InChIKey) with (1) the published homologous series and (2) published structure category CSV files respectively. Then, the merged data were manually inspected. The script and all CSV files resulting from this validation analysis are available in the Additional file 1: Sect. 3.

To compare OngLai to an existing method for categorising PFAS compounds called splitPFAS, OngLai was additionally applied to the 770 PFAS listed in the Supplementary Information file of Sha et al. [53] Homologous series with CF_2_ repeating units detected by OngLai in NORMAN-SLE were compared with the categorisation results of splitPFAS. In the original paper, 770 PFAS were systematically divided into 4 categories with general formulae C_n_F_2n+1_-X-R: perfluoroalkanoyl (X = CO), sulfonyl (X = SO_2_), n:1 fluorotelomer (X = CH_2_), and n:2 fluorotelomer (X = CH_2_CH_2_). For comparison purposes, compounds with the same X and same R groups but differing *n* are considered to form homologous series (henceforth referred to ‘splitPFAS series’). Python code used to prepare and analyse the splitPFAS dataset and all results from the comparative analysis are available in Sect. 4 of Additional file 1.

## Results and discussion

OngLai was applied to 3 different datasets by running the Python script in the command line within a conda environment containing the RDKit. The script and all necessary modules are provided in the OngLai package on GitHub (see https://github.com/adelenelai/onglai-classify-homologues for the full list). A compute server with two Intel(R) Xeon(R) Silver 4114 CPUs and 64 GB of RAM was used in single-thread mode. OngLai’s default settings (Table 2) were applied, including using ‘[#6&H2]’ corresponding to CH_2_ (alkyl) as the repeating unit SMARTS input (Table 1). Detection of homologous series by OngLai in NORMAN-SLE, PubChemLite, and COCONUT datasets using these parameters took approximately 2, 16, and 35 min respectively. Two further runs of the algorithm were performed on each dataset using ‘[#8]-[#6&H2]-[#6&H2]’ and ‘[#6](-[#9])(-[#9])’ as repeating unit SMARTS input, corresponding to CCO (ethoxy) and CF_2_ (perfluoroalkyl) respectively; for validation, the homologous series detected in the NORMAN-SLE dataset were compared to the published lists as described above. Additionally, OngLai was also run on the 770 PFAS compounds used in the splitPFAS study for comparison.

This section is divided into two parts. First, an overview of the homologous series with CH_2_ repeating units classified in the three datasets is provided, including an interpretation of OngLai’s outputs, validation of the CH_2_, CCO and CF_2_ series classified in NORMAN-SLE, and comparison with splitPFAS. Then, the second part focuses on the implementation and behaviour of OngLai’s underlying algorithm, demonstrated in detail using selected examples of classified homologous series.

### Homologous series classified in NORMAN-SLE, PubChemLite, and COCONUT

**Fig. 3:**
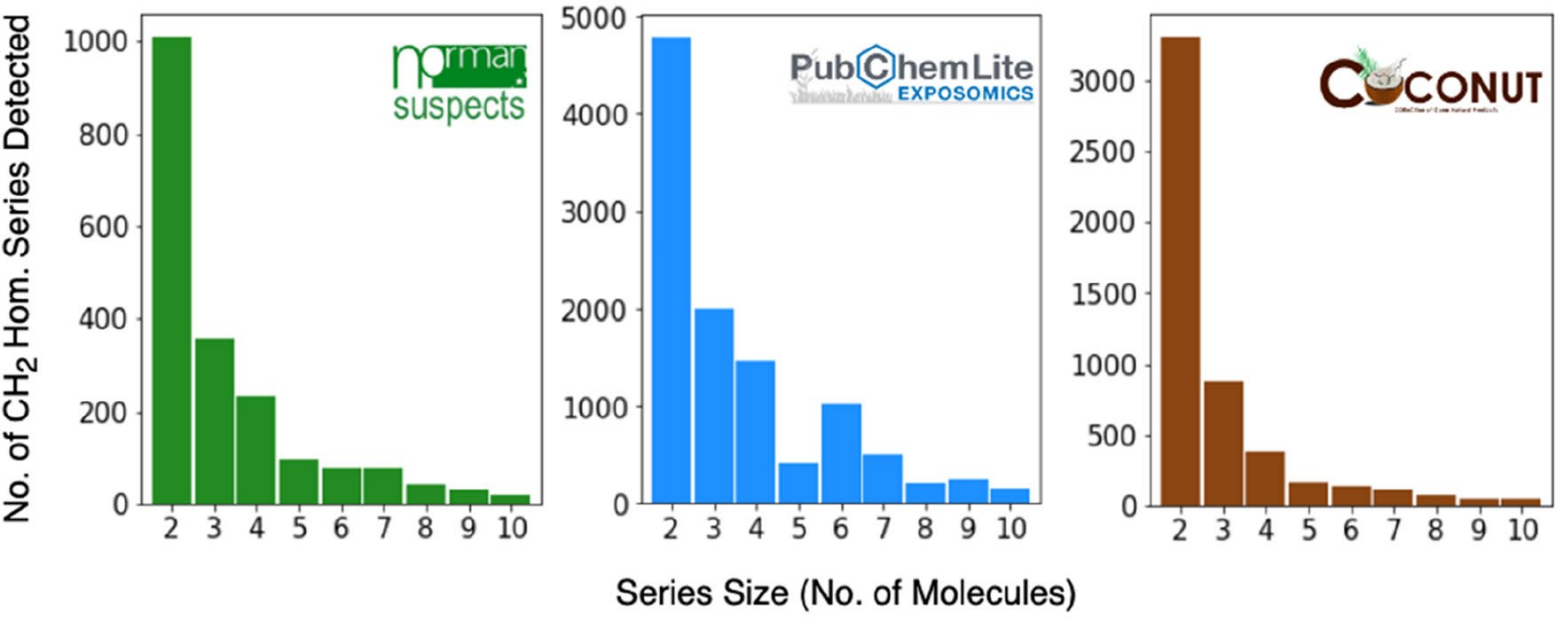
Numbers of homologous series with CH_2_ repeating unit detected within the three datasets, sorted by series size (only series sizes up to 10 molecules shown here). The algorithm’s default settings were used, as listed in Table 2

Thousands of homologous series with CH_2_ repeating units were detected by OngLai: in total, 2098 in NORMAN-SLE, 12,105 in PubChemLite, and 5329 in COCONUT respectively. These series were detected using the default settings of the algorithm (Table 2). The size distributions of the homologous series classified are shown in Fig. 3, while Table 4 provides a summary of the overall results. Complete series classification results are available in Sect. 2 of the Additional file 1. Notably, most series detected comprise only 2 molecules, similar to chemical series classified within drug discovery projects [68]. Overall, there are more small series than there are large series, as evident in the series size distributions (Fig. 3), which may imply a high chemical diversity in the respective databases.

**Table 4:** Summary statistics of detected homologous series with CH_2_ repeating units in the three datasets. The algorithm’s default settings were used, as listed in Table 2. Full details and results are available in Additional file 1: Sect. 2

	NORMAN-SLE (n = 98,116)	PubChemLite (n = 392,465)	COCONUT (n = 407,270)
No. of homologous series detected	2098	12,105	5329
No. of molecules classified as members of homologous series	8775	82,476	18,528
No. of molecules consisting purely of CH_2_ repeating units	0	0	0
No. of molecules containing CH_2_ repeating units but not forming homologous series (unique cores)	10,778	35,111	36,864
No of molecules not containing CH_2_ repeating units	78,559	274,861	351,527
No of molecules discarded from analysis (failed sanitation)	4	17	351

The proportion of molecules that were deemed members of CH_2_ homologous series given the default settings used were 9% for NORMAN-SLE, 21% for PubChemLite, and 5% for COCONUT (Table 4). Approximately 10% of each dataset consists of molecules that contain CH_2_ repeating units, but do not form homologous series, meaning the detected cores are unique within the respective dataset. The majority (70–86%) of all molecules in each dataset do not contain CH_2_ repeating unit chains of minimum length 3 repeating units (Table 2, default algorithm setting), i.e., there were no substructure matches found in those molecules using the following SMARTS query: ‘[#6&H2]-[#6&H2]-[#6&H2]’, representing the structure ‘CH_2_CH_2_CH_2_’ in pseudo-SMILES. Overall, less than 5% of molecules were discarded from the analysis because they were either not parseable by the RDKit due to valence model violations e.g., pentavalent carbons, or the SMILES strings were invalid (reported to the respective data maintainers).

Notably, zero molecules consisting purely of CH_2_ repeating units were detected across the three datasets. Instinctively, one would think alkanes such as propane, butane, and pentane fall into this category, but they do not because the terminal carbon atoms in these alkanes are bonded to three H atoms and not exactly two, as specified in the SMARTS representing CH_2_ repeating units (Table 1, ‘[#6&H2]’). Therefore, alkanes are considered to form their own homologous series by OngLai, with the terminal carbon atoms ultimately forming the core (‘H_3_C. CH_3_’ in pseudo SMILES). This result highlights how the specificity of the SMARTS repeating unit definition directly determines the homologous series classified, which is further discussed in “Effect of repeating unit SMARTS specification on homologous series classified”.

Details of the CCO and CF_2_ homologous series detected in the three datasets are available in Additional file 1: Sect. 2. Notably, 64 molecules in COCONUT were classified into 23 homologous series with CF_2_ repeating units. These molecules do not appear to be natural products and should be removed in future curation exercises of natural product space. As these molecules have been classified into series, entire series of these non-natural-product-like molecules can be removed together instead of having to search and remove these molecules on an individual basis. These findings have been reported to the COCONUT database maintainers [69].

### Validation of classified series

The validation of homologous series classified in NORMAN-SLE was performed in two ways: (1) by comparing classified series with published homologous series, and (2) by inspecting their homologous compound membership within published structure categories. All validation results described below are available in Sect. 3.3 of Additional file 1.

### Validation with published homologous series

**Fig. 4:**
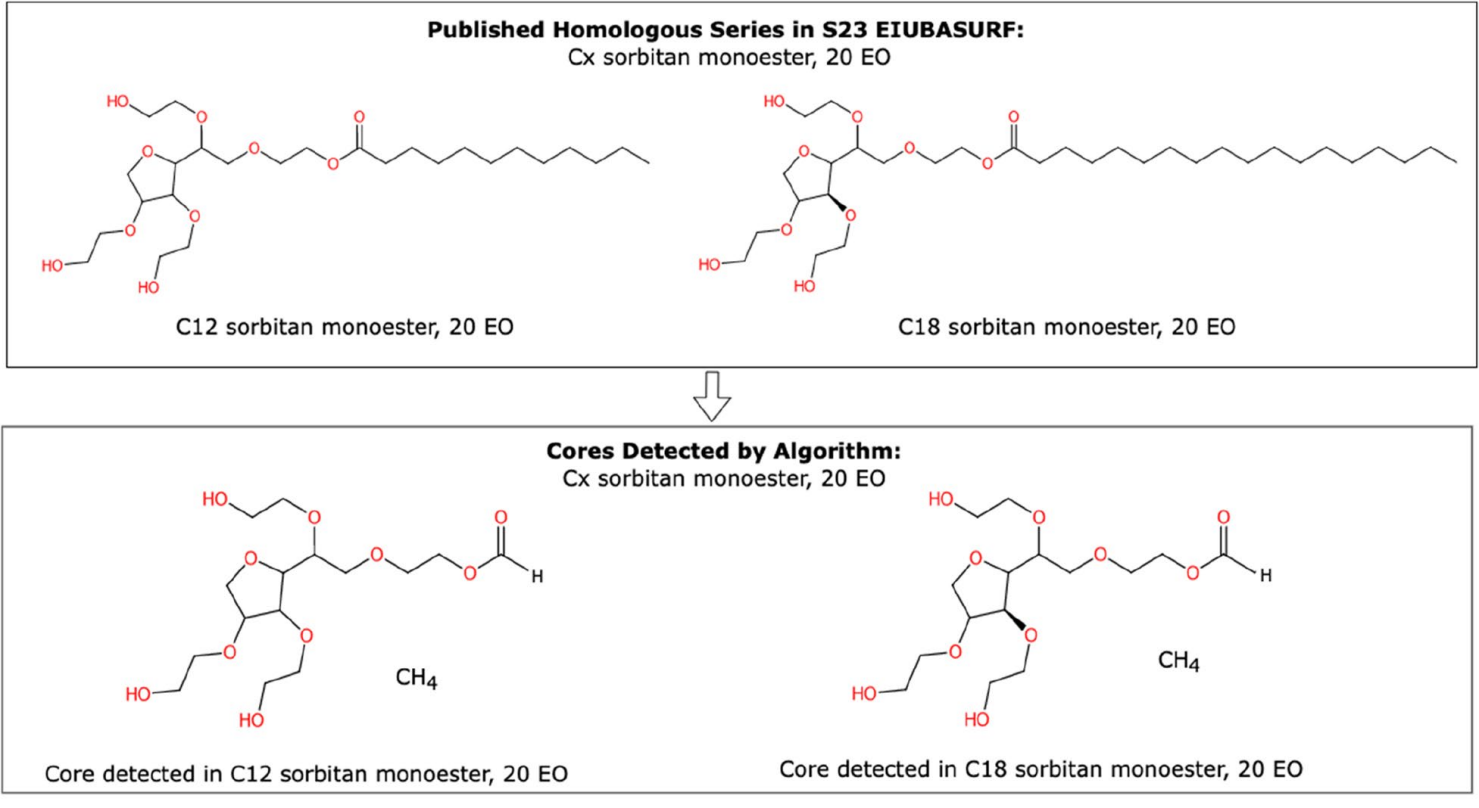
Example of 2 homologous molecules that were not classified as a series (CH_2_ repeating units) by OngLai. The ‘Cx sorbitan monoester, 20 EO’ series is published in *S23 EIUBASURF*, but was not classified by the algorithm because of differing stereochemistry within the NORMAN-SLE dataset, and therefore different cores detected. Full details are in Additional file 1: Sect. 2

As shown in Table 5, the majority of CH_2_ and CCO homologous series detected in NORMAN-SLE were in overall agreement with published homologous series in *S7* and *S23* (62%, 60%, 80%, 64% ‘Full Match’ respectively). Partial or mixed classifications arose due to various factors such as suboptimal algorithm settings for that particular series of molecules (e.g., the minimum repeating unit chain length of 3 was too long), or differences in stereochemistry specificity across molecules that would otherwise belong to the same series within NORMAN-SLE. Less than 5% of homologous series were not identified by OngLai across all repeating units and published homologous series because of either of the two aforementioned factors. An example of published homologous molecules that were not classified by OngLai is the ‘Cx, sorbitan monoester, 20 EO’ series. This series is listed in *S23 EIUBASURF* as having two molecules (x = 12 and 18). In the NORMAN-SLE dataset however, the C_12_ species has no stereochemistry specified, but the C_18_ species does, thus causing them to have different cores detected, resulting in the series not being classified by OngLai (Fig. 4; further discussion on stereochemistry below). In this sense, OngLai provides a more specific classification of homologous series than what is listed and indicated by the Name field in *S23 EIUBASURF*, as it distinguishes between levels of stereochemical information specificity that were not captured by the naming convention used in *S23 EIUBASURF*.

**Table 5:** Validation by comparing homologous series in NORMAN-SLE classified OngLai with published homologous series containing CH_2_ and CCO repeating units. Series in S7 and S23 were manually compared to OngLai results. Full Match indicates a 1:1 relationship between published series and series classified by OngLai. Homologous series from NORMAN-SLE containing molecules that are not in the published homologous series list or vice versa, but that otherwise match, are also considered Full Matches (‘or as available’). Partial or Mixed Classification indicates either a 1:n relationship between published homologous series and homologous series classified by the algorithm, or that certain molecules were not classified together with the others in a given published series. Full details in Additional file 1: Sect. 3.3

List containing published homologous series	Repeating unit	No. of published homologous series				
		Full match (or as available)	Partial or mixed classification	Not classified by OngLai	Present in list, absent in NORMAN-SLE	Total
S7 EAWAGSURF	CH_2_	8	5	0	0	13
	CCO	6	4	0	0	10
S23 EIUBASURF	CH_2_	105	17	6	4	132
	CCO	62	35	0	0	97

Importantly, validation using published homologous series in the *S7* and *S23* datasets was possible because of the naming convention used by the datasets’ curators. For example, in these datasets, compounds with the names C9-LAS, C10-LAS, C11-LAS, and C12-LAS clearly belong to the Cx-LAS series. The fact that homologous compounds in these datasets can be recognised just from their names without any inspection of their chemical structures supports the use of these lists as independent sources of information ideal for homologous series validation.

### Validation with published structure categories

Similar results were obtained in the validation of classified homologous series with CF_2_ repeating units using the OECD’s PFAS Structure Categories: 50% of the 600 homologous series detected contain molecules that belong to the same single Structure Category within the respective series (Table 6). The remainder corresponds to homologous series containing molecules belonging to more than one Structure Category (10% of all series classified), no Structure Category (22.5%), or a mixture thereof (17.5%) within the same series.

**Table 6:** Comparison of published Structure Categories for PFAS compounds containing CF_2_ repeating units with homologous series classified by OngLai in the NORMAN-SLE dataset. Structure categories are published in the 2018 OECD PFAS report [52, 67]

	Series with 1 structure category	Series with > 1 structure category	Series with no structure category	Series with combination of no structure category and ≥ 1 structure category	Total series classified by OngLai
No. of CF_2_ homologous series	301	59	135	105	600

**Fig. 5:**
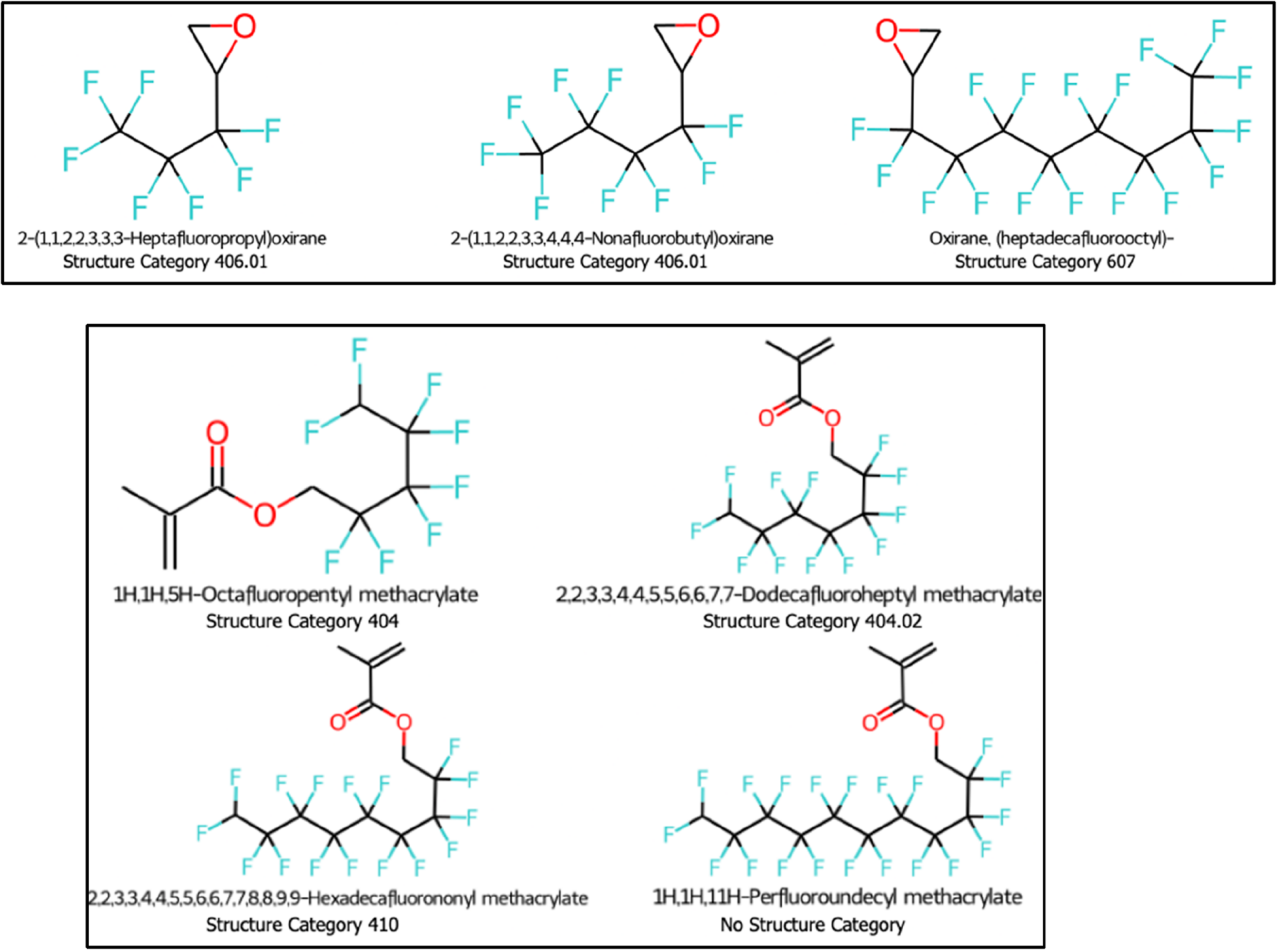
Examples of classified homologous series with CF_2_ repeating units composed of molecules belonging to different OECD Structure Categories. Molecules from the NORMAN-SLE dataset (series_no = 11 and 13, Additional file 1: Sect. 2)

Two examples of molecules grouped into the same series having different OECD Structure Categories are shown in Fig. 5. The molecules in the first series (Fig. 5, top panel) belong to two different Structure Categories: Category 406.01 corresponding to fluorotelomer epoxides (CnF2n + 1I + CH2 = CHCH2OH – > CnF2n + 1-CH2CH(I)CH2OH – > CnF2n + 1-CH2(CHCH2O)); and Category 607 corresponding to perfluoroalkyl epoxides & derivatives (CnF2n + 1-epoxides). Another example (Fig. 5, bottom panel) has molecules in the same classified series that do not belong to any Structure Category *and* a combination of Structure Categories 404 − n:1 fluorotelomer-based non-polymers (CnF2n + 1-CH2-R); 404.02 − n:1 FT (meth)acrylate (CH2–OC(=O)CH=CH2); and 410 − n:1 FT (meth)acrylate (CH2-OC(=O)CH=CH2). The last molecule in the series does not belong to any OECD Structure Category because it is absent from the original *S25 OECDPFAS* list, but was present in the NORMAN-SLE because it originated from other lists (e.g.,* S46* and *S71)* that make up the PFAS within NORMAN-SLE.

These mixed results are attributable to the broader definitions of Structure Categories compared to homologous series; the former often contain a mixture of homologous and non-homologous molecules. Per the 2018 OECD definition, a Structure Category can represent various properties, such as sharing a common general formula, varying functional groups, and/or being derivatives of the same compound e.g., ‘category 101: perfluoroalkyl carbonyl halides (CnF2n + 1-C(= )R, R =F/Cl/Br/I)’ and ‘category 202: perfluoroalkane sulfonic acids (PFSAs), their salts and esters (R =OH, ONa, OCH3, etc.)’. These relatively broader categories likely reflect some of the challenges of assigning Structure Categories to numerous PFAS in a manual fashion, as was done for the 2018 OECD PFAS definition. As manual assignment is prone to typographical errors, wrong assignments, or inconsistent assignments, cheminformatic-based tools for automated assignment of Structure Categories are highly desirable and warranted [53, 70, 71].

Overall, as approximately 50% of CF_2_ series classified by OngLai in the NORMAN-SLE dataset contain molecules belonging to the same OECD Structure Category, there appears to be reasonable consistency in the 2018 OECD manual categorisation of PFAS. Given that the homologous series classified by OngLai have stricter definitions in terms of chemical structure similarity, OngLai’s results could support or inform future OECD efforts to subcategorise PFAS.

### Comparison with existing method for categorising PFAS: splitPFAS

OngLai was applied using the same compute server described above to the 770 PFAS compounds that were originally categorised by splitPFAS. In approximately 2 min, 132 homologous series with CF_2_ repeating units were classified (Table 7). These results were compared with those of the splitPFAS tool (XLSX file in Supplementary Information of Sha et al. [53]). For comparison here, molecules in a given PFAS category out of the four outlined by Sha et al. that share identical R groups are assumed to be homologous series because they have the same general formula (same X and R groups in C_n_F_2n+1_-X-R). These series will henceforth be referred to as ‘splitPFAS series’. There were 124 of such splitPFAS series found in Sha et al.’s work; OngLai detected 132 homologous series (full details in Sect. 4 of Additional file 1).

**Table 7:** Summary statistics of detected homologous series with CF_2_ repeating units in the splitPFAS dataset

	splitPFAS dataset (n = 770)
No. of series detected	132
No. of molecules classified as members of homologous series	540
No. of molecules consisting purely of CF_2_ repeating units	0
No. of molecules containing CF_2_ repeating units but not forming homologous series	196
No. of molecules not containing CF_2_ repeating units	34
No. of molecules discarded from analysis (failed sanitation)	0

**Fig. 6:**
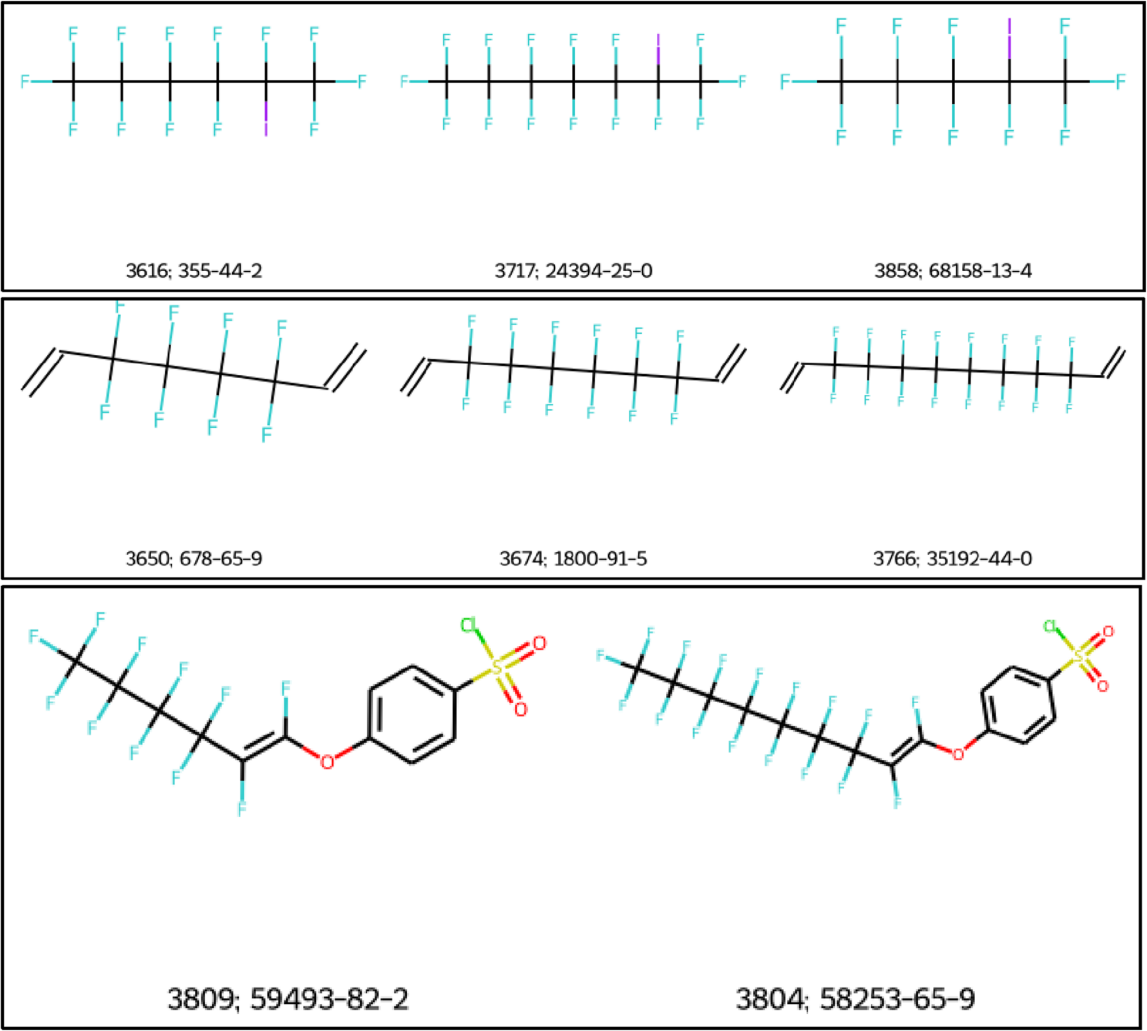
Examples of homologous series classified by OngLai that were not categorised by splitFAS because ‘No splittable bond [was] found’. Labels correspond to the ‘ID_in_OECD_list’ and ‘CAS’ fields given in the splitPFAS XLSX results file respectively; molecules from the splitPFAS dataset (series_no = 10, 109, and 116, Additional file 1: Sect. 2)

Comparison of the series classified by OngLai and splitPFAS series generally shows good agreement between the two methods in terms of their matching results. However, there are some differences in the number of series and composition of certain series which can partly be attributed to the fact that some PFAS were not categorised by splitPFAS, but were classified as homologous series by OngLai. The reason for this result is because within splitPFAS outputs, no X groups were detected for these molecules by splitPFAS. Consequently, in the results XLSX file, these molecules have ‘NA’ in their ‘SplitSMARTS (X)’ column, attributed to ‘No splittable bond found for the input molecule’. Associated error codes provided as splitPFAS output explain the various underlying reasons, for example ‘1—the perfluoroalkyl chain was branched or cyclic’, or ‘4—the R group was a single F atom’. There were 11 homologous series classified by the algorithm containing such molecules (examples in Fig. 6).

**Fig. 7:**
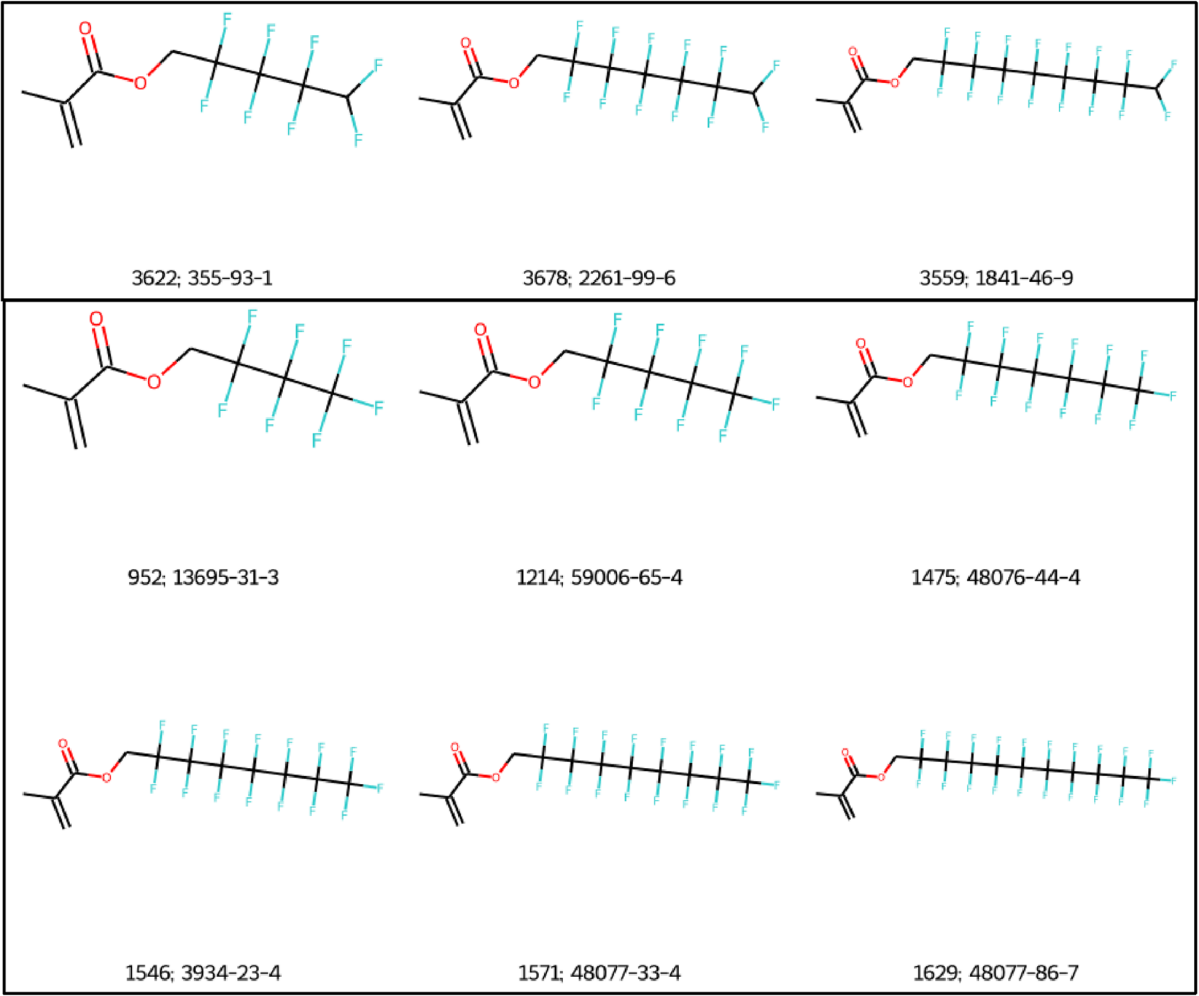
Examples of two series classified by OngLai that belong to the same splitPFAS series because they have the same X and R groups in C_n_F_2n+1_-X-R according to splitPFAS results. Labels correspond to the ID_in_OECD_list and CAS Registration Number given in the splitPFAS results XLSX file respectively; molecules from the splitPFAS dataset (series_no = 0 and 1, Additional file 1: Sect. 2)

Another reason for the difference in the results produced by splitPFAS and OngLai is that some PFAS do not actually conform to the general formula C_n_F_2n+1_-X-R prescribed by Sha et al. For example, all the molecules shown in Fig. 7 have the same X groups and R groups in the general formula prescribed by Sha et al. (C_n_F_2n+1_-X-R), as indicated in the splitPFAS results (XLSX file, Fluorotelomer tab), where X=[CH2] and R=CC(=C)C(=O)O (methylacrylic acid). Therefore, they technically belong to the same splitPFAS series according to the assumption made for this comparison exercise. Evidently however, the molecules in the top panel of Fig. 7 actually have the general formula C_n_F_2n_-X-R because the terminal carbon is bonded to two fluorine atoms and one hydrogen atom instead of three fluorine atoms, as in the bottom panel. In this case, OngLai distinguished this fact; the core detected for the series in the top panel of Fig. 7 is methacrylate, while that for the series in the bottom panel consists of two disconnected fragments: methacrylate and a fluorine atom. As shown in this example, OngLai was able to distinguish and thus group different PFAS into homologous series with higher granularity than splitPFAS.

Overall, the categorisation results of splitPFAS are very similar to the results of the presented homologous series classification algorithm (full results available in Additional file 1: Sect. 4). This outcome indicates that the assumption made for the purpose of this comparison—that compounds having the same X and R groups in the general formula C_n_F_2n+1_-X-R are indeed homologous—was reasonable. However, in some cases, OngLai demonstrated more flexibility in handling different PFAS structures than splitPFAS because the latter has more hard-coded elements in its cheminformatics processing of input structures than OngLai does. For example, splitPFAS has specific SMARTS corresponding to the 4 PFAS categories specified, which likely explains why no splittable bonds could be detected in some cases. That said, it is important to bear in mind that splitPFAS was designed with a different intention than OngLai; splitPFAS is not dedicated to homologous series classification, therefore it cannot be directly compared. Nevertheless, this comparison shows that OngLai could be used to support PFAS categorisation efforts by e.g., providing further subcategorisation.

### Implementation of OngLai

In this section, important features of the OngLai algorithm and its implementation, independent of the datasets it is applied to, are discussed using demonstrative examples of CH_2_ series classified across NORMAN-SLE, PubChemLite, and COCONUT.

### Molecular fragmentation—removing one substructure match at a time

**Fig. 8:**
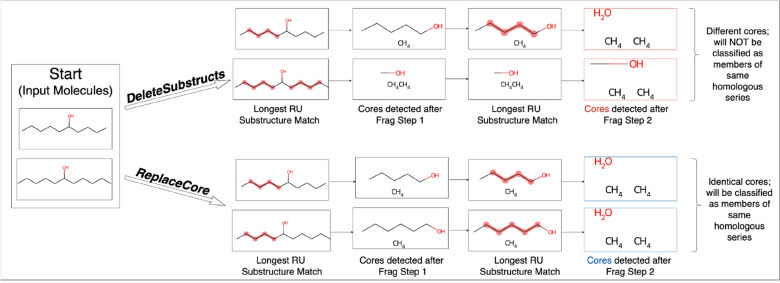
The impact of different fragmentation approaches in the RDKit on homologous series core detection, top: using *DeleteSubstructs*, bottom: using *ReplaceCore*. The two input molecules are homologous and should be classified into the same series; fragmentation using *ReplaceCore* achieves this as identical cores are detected (C.C.O in SMILES representation). However, *DeleteSubstructs* yields different cores (C.C.O and C.C.CO in SMILES respectively) for the two input molecules because both –CH_2_–CH_2_–CH_2_–CH_2_– chains of the symmetrical molecule are removed simultaneously in Fragmentation Step 1, resulting in inequivalent cores and no homologous series detected

In cheminformatics, removing one substructure match at a time instead of multiple simultaneously in a given molecule is not a trivial task, yet here, it is crucial for preserving the accuracy of the core detected and thus correct classification of homologous series. In the RDKit, the most intuitive choice to achieve substructure removal is *DeleteSubstructs,* but this function removes all repeating units matched at a time in one go, which is undesirable. Therefore, *ReplaceCore* is used instead and shown in comparison to *DeleteSubstructs* in Fig. 8. To date, the RDKit community has explored two further alternatives to remove one substructure at a time [72], but these methods are not suitable here because (1) there is no way to remove entire substructures from *RWMol* objects, only atoms and bonds, and (2) encoding the substructure to be removed as a chemical reaction is impractical, as a new Reaction SMARTS query would have to be encoded for each input molecule depending on its specific structure. In this sense, *ReplaceCore,* typically used for common cheminformatic tasks like R-group decomposition or constructing Structure–Activity Relationship tables*,* was applied here in a novel and perhaps unorthodox, but effective manner to remove substructures.

### Effect of repeating unit SMARTS specification on homologous series classified

**Fig. 9:**
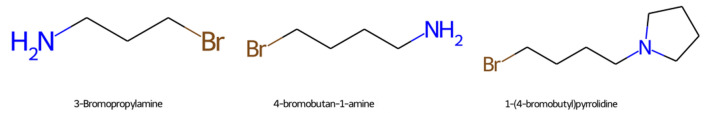
Example of a CH_2_ series containing members where the alkyl repeating units were found within a ring and a linear chain. Molecules from PubChemLite (series_no = 30, Additional file 1: Sect. 2)

As described in a previous example in this section, the repeating unit SMARTS definition directly influences the homologous series classified, for example, by explicitly defining the exact number of connected hydrogen atoms. Other properties of atoms defined in the SMARTS string also play an important role: in the default repeating unit SMARTS used, ‘[#6&H2]’, the carbon atom is bonded to exactly two hydrogen atoms, regardless of that carbon’s ring membership. Therefore, repeating units forming rings would also be positive matches just like repeating units in linear chains, as shown in Fig. 9, where the CH_2_ moieties in the pyrrolidine ring of 1-(4-bromobutyl)pyrrolidine hydrobromide, in addition to those in the linear chain, matched the repeating units SMARTS ‘[#6&H2]’. Thus, these matches were subsequently removed during molecule fragmentation in the core detection process. The resultant core common to all these three molecules thus consists of two disconnected atoms, one bromine and one nitrogen (‘Br.N’ in SMILES).

However, if a more specific repeating unit SMARTS query specifying ring membership is used, the first two molecules could be distinguished from 1-(4-bromobutyl)pyrrolidine. Using the repeating unit ‘[#6;!R&H2]’ (carbon atom that is not a member of a ring bonded to exactly two hydrogen atoms) yields two different cores for the three molecules in Fig. 9: while the core detected for the first two molecules remains the same as before, that for 1-(4-bromobutyl)pyrrolidine consists of the intact pyrrolidine ring and a single Br atom, represented in SMILES as ‘Br.C1CCN(C1)’. Thus, 1-(4-bromobutyl)pyrrolidine would not be included in the same homologous series as the first two molecules in Fig. 9 which underscores the importance of repeating units SMARTS specification in the resulting homologous series classified. In other words, users should be careful when specifying their repeating units SMARTS to achieve the desired results.

### Effect of maximum length of repeating unit chains specified

**Fig. 10:**
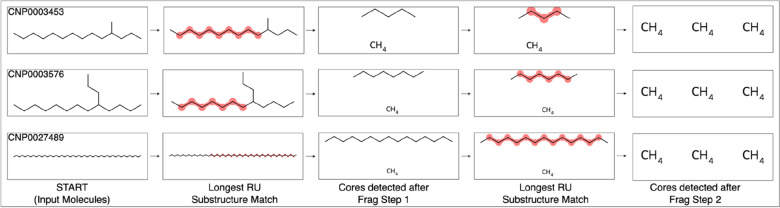
Example of three molecules classified into the same homologous series with CH_2_ repeating units. The longest repeating unit chain in the bottom molecule CNP0027489 is longer than the maximum repeating unit chain length specified as algorithm input (30 units), resulting in only partial removal after Fragmentation Step 1. Subsequently, three core fragments were detected instead of two. Molecules from COCONUT dataset (series_no = 126, Additional file 1: Sect. 2)

The maximum length of repeating unit chains to be enumerated for substructure matching and removal is user-customisable, with the default value set to 30 repeating units (Table 2). This default value was used in the present analysis to avoid prolonged computation times that result from having a larger maximum value. It was also assumed that this would be sufficient to cover all possible cases of repeating units in the molecules analysed. This assumption held true for the NORMAN-SLE and PubChemLite datasets, but not COCONUT, where some molecules were misclassified due to this default value (see Fig. 10).

In the classified homologous series shown, the linear alkane CNP0027489 (molecular formula C_46_H_94_) should have been classified together with other linear alkanes having core (‘CH_3_. H_3_C’ in pseudo SMILES). However, because the longest repeating unit chain in CNP0027489 is C_44_H_88_ (corresponding to a maximum repeating unit length of 44) and not C_30_H_60_ (a maximum repeating unit length of 30), the resulting core after two fragmentation steps contains three CH_3_ fragments instead of two, causing it to be classified together with branched alkanes having the same core. In this case, correct classification would be achieved if the maximum value was set to 44 or higher, albeit at the expense of significantly longer computational times.

### Effect of number of fragmentation steps

The ‘No. Fragmentation Steps’ setting (Table 2) affects the extent of fragmentation of the input molecule and as a result, the cores detected. Therefore, the cores detected can vary in structure depending on the number of fragmentation steps specified, especially in cases where (1) there are multiple repeating unit chains within a given molecule, (2) the repeating unit chains are of different lengths, and/or (3) the repeating unit chains are bonded to the same atom.

**Fig. 11:**
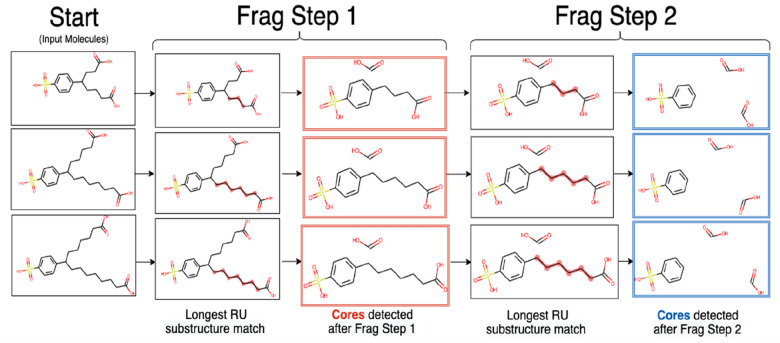
The number of fragmentation steps given as input affects core detection, shown here with three dicarboxylated alkyl benzenesulfonate molecules originally from NORMAN-SLE. These compounds are detected frequently in environmental samples as transformation products of commonly used surfactants [27]. Note that the core fragments in the red and blue boxes are depicted in their sanitised forms without dummy atoms to reflect the SMILES they would be grouped together by

Figure 11 shows the impact of varying the number of fragmentation steps on three input molecules belonging to the same homologous series ‘Cx-SPADCs’, published in *S7 EAWAGSURF*. Starting with the input molecules in the left-most column, had ‘No. Fragmentation Steps' been set to 1, the final cores detected would have been those shown in the red boxes. However, as none of these cores are identical to each other, these three molecules would not be classified into the same homologous series. In contrast, a second fragmentation step yields identical cores for the three input molecules (Fig. 11 blue boxes) that would result in the three input molecules being grouped together into the same series. Thus, the number of fragmentation steps selected is crucial for appropriate core detection and homologous series classification.

### Effect of sanitisation on core detection

**Fig. 12:**
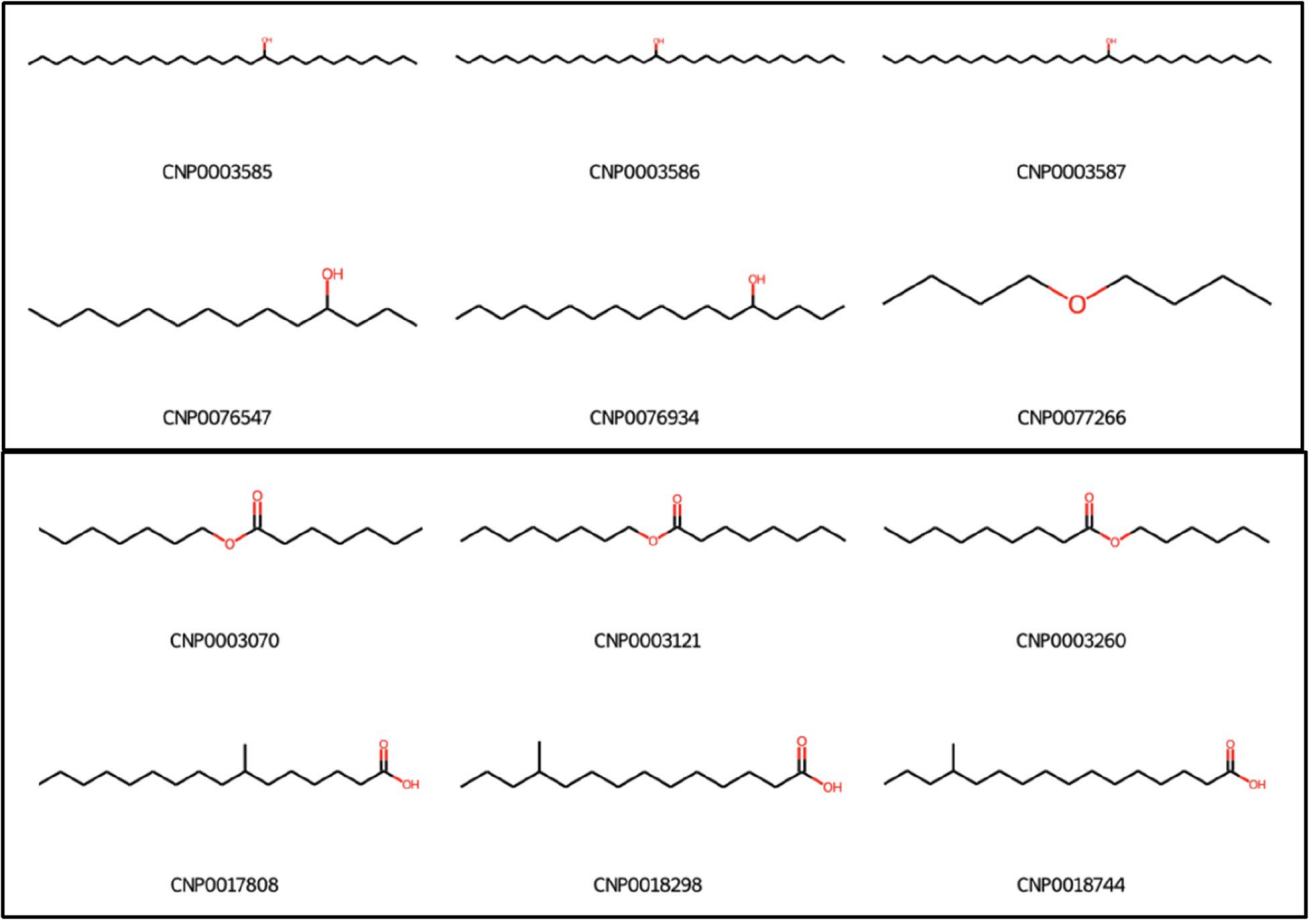
Examples of CH_2_ series containing members with cores in different positions within the molecule. Top: alcohols and ethers have the same core (C.C.O in SMILES), bottom: carboxylic acids and esters (C.C.O = CO in SMILES). Molecules are from COCONUT, and selected members from each series are shown (series_no = 597 and 662, Additional file 1: Sect. 2)

The position of core fragment(s) within input molecules is irrelevant for OngLai. In other words, molecules containing the same core fragments, albeit in different positions within the molecule relative to the repeating units, are classified into the same homologous series. Concrete examples are shown in Fig. 12, where molecules containing either alcohol or ether functional groups are considered homologous (Fig. 12, top panel). A second example shows molecules containing either a carboxylic acid or ester moiety belonging to the same classified series (Fig. 12, bottom panel). Here, whether the core is in a terminal or central position within the molecule is not considered in core detection because its atomic neighbourhood is not taken into account. Consequently, the number of repeating unit chains attached to the core is also not considered, meaning the core could be attached to carbons of varying connectivity degrees across the different members of a homologous series. For example, the ‘O’ fragment in the ether core of molecule CNP0077266 is attached to two primary carbon atoms (Fig. 12, top panel), while the ‘O’ fragments in the other molecules of the same series shown are attached to one secondary carbon atom each. Depending on user preference, grouping together molecules with varying core fragment position in the same homologous series may be desirable, but it is possible that future augmentations of OngLai could address the consideration of the number of repeating unit chains attached to the core, or atomic neighbourhood of the core in general.

### Effect of stereochemical information

**Fig. 13:**
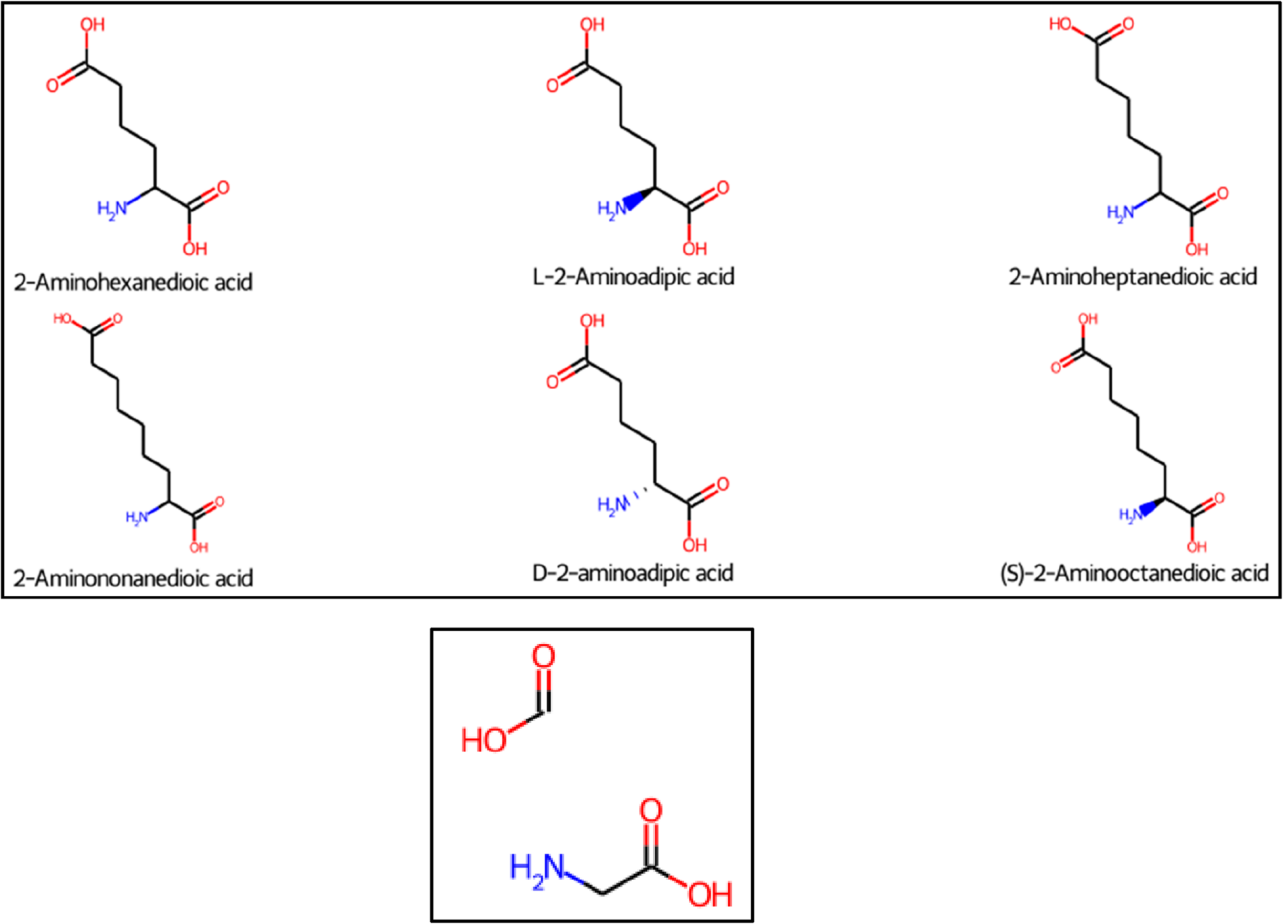
Example of CH_2_ series containing molecules with stereochemical information. The core of this series (bottom panel) has stereochemistry removed in the process of core detection, hence all the molecules in the top panel are considered homologous despite their various stereochemistries. Molecules from NORMAN-SLE (series_no = 2021, Additional file 1: Sect. 2)

Stereochemical information can play a discriminatory role in homologous series detection, depending on where it is specified relative to the core fragment(s) and molecular fragmentation site(s). If bonds with no stereochemistry specified connecting repeating units and core fragments are fragmented, but stereochemical information is present elsewhere in the molecule, the latter is preserved and taken into consideration during the process of homologous series detection via grouping molecules with identical cores. For example, as shown in Fig. 4, the ‘C_18_ sorbitan monoester’ input molecule contains a bond pointing outwards, as does its core. However, the ‘C_12_ sorbitan monoester’ and its core have planar bonds throughout, so the C12 and C18 species are not considered homologous by OngLai. In contrast, the molecules in Fig. 13 are classified as homologous despite their different stereochemistries, because the amino acid core fragment common to all 6 molecules (Fig. 13, bottom panel) was originally adjacent to the fragmented bond and therefore experienced stereochemistry neutralisation in the process of core detection (addition of dummy atom, then conversion to hydrogen atom). Thus, molecules with different stereochemistries may be grouped into the same series if fragmentation happens on bonds or adjacent bonds that originally have stereochemistry specified, as this information is removed during core detection. This behaviour is desirable in the specific case of annotating databases to support the identification of chemicals in environmental samples using mass spectrometry (which was the original motivation of OngLai), where stereochemistry differences are less relevant for compound identification. By grouping together all homologous compounds regardless of their stereochemistry differences, the remaining ‘unannotated’ chemical space that should be considered for unknown identification would be smaller, which could make unknown identification easier and more efficient. Overall, however, the desirability of this behaviour would depend on the individual user’s ultimate goal and intended application of classifying homologous series.

Regarding stereochemistry in the datasets used relative to their preparation as described in “Methods”, only the molecules in COCONUT have no stereochemistry encoded, whereas molecules in NORMAN-SLE and PubChemLite have mixed stereochemical information availability. To investigate the influence of stereochemistry on homologous series detected further, future efforts could include applying OngLai to the version of COCONUT containing all stereoisomers.

### Molecules with branched repeating units classified as series

**Fig. 14:**
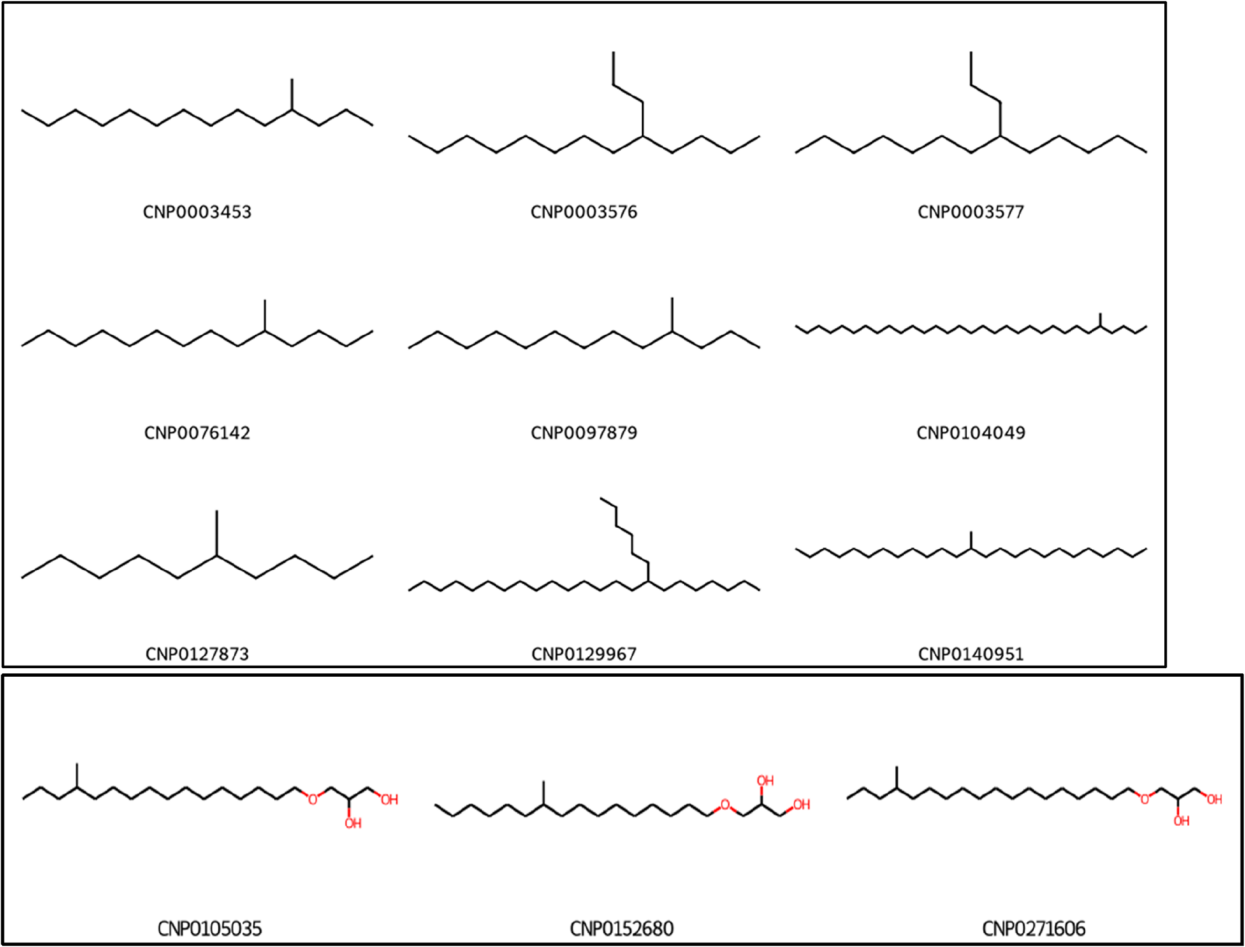
Example of two CH_2_ series containing molecules with varied branched repeating units. Molecules from COCONUT (Bottom: series_no = 696; top: selected 9 of total 78 molecules in series_no = 126, Additional file 1: Sect. 2)

Molecules with branched repeating units, irrespective of the length of the branch and branching site, are classified into the same series since OngLai does not consider the atomic neighbourhood of the matched repeating units it removes during core detection (Fig. 14). Rather, it simply detects the longest repeating unit chain and removes it in the process of series classification. In certain applications, this insensitivity could be advantageous, for example when characterising chemical space or preparing diversity decks in high-throughput chemical screening [35, 73], as grouping together such highly similar analogues could result in reduced redundancy and better representation of the molecules within a given chemical series. However, it is also possible that this insensitivity to the site and extent of branching could be addressed in future augmentations of the algorithm by e.g., introducing filters for molecules that have repeating unit chains of the same lengths.

### Structural isomers classified as series

**Fig. 15:**
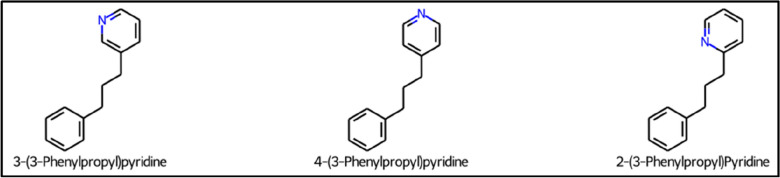
Example of a CH_2_ series containing structural isomers. The homologous molecules in this series have cores with ring substituent positions in the meta-, para- and ortho-positions respectively going from left to right. Molecules from NORMAN-SLE. (series_no = 2097, Additional file 1: Sect. 2)

As explained above, the atomic neighbourhood of repeating units is not considered when repeating units are being matched for substructure removal in core detection. Thus, being insensitive to atomic neighbourhoods results in ring substitution isomers (meta-, para-, and ortho-) being classified as members of the same series, irrespective of the attachment position of the repeating unit chain (Fig. 15). If desired, such occurrences could be identified and filtered or grouped together on the basis of formula or mass in a post-processing step.

## Future work

The present work introduces OngLai, an algorithm to classify homologous series within compound datasets. Since this topic has been relatively unexplored, three areas of further research could be interesting to pursue based on the work presented here. Additionally, integration of this homologous series classification functionality into existing tools such as the ‘Contrib’ directory of the RDKit and the R package ‘patRoon’ [74] to further enhance the utility of this algorithm have already been discussed with the relevant software maintainers.

### Algorithm

Consideration of the atomic neighbourhood of the core fragment(s) during core detection is a potential feature to implement in the next version of OngLai. As highlighted in the “Discussion” section, doing so could improve the accuracy of core detection and thereby generate homologous series containing molecules that have less variability with respect to branching, structural isomerism, and position of the core in the molecules. Atomic neighbourhood consideration could be achieved by attaching R-groups onto repeating unit chains at the fragmentation site, then integrating this information when grouping identical cores together in the final step of homologous series classification.

### Further results analysis

Additional automated analyses can be performed with homologous series structures after their classification in a given dataset. A first functionality could be to order the series by the number of chains and the number of repeating units in their chains within one identified homologous series. Alternatively, homologous series could be grouped together based on multiple characteristics or properties such as having the same type of repeating units or similar core fragments, e.g., homologous series with core fragments that represent different ortho/meta/para variants of the same structure could be grouped. At higher levels, classified series could be grouped according to similarities between their core or repeating units, based on a defined similarity measure. Basically, any known chemical clustering algorithm can be applied to representative structures of each homologous series group here. This grouping and ordering for different characteristics at different levels can result in a homologous series hierarchy for the given dataset, similar to a scaffold tree [75], which could allow for an intuitive, multi-layer visualisation of homologous series diversity in a given dataset. In terms of mass spectral data processing, specific groups of homologues of interest could also be used either as potential suspect lists or database files during non-target LC-HRMS data processing.

### Alternative cheminformatics approaches to classify homologous series

Currently, repeating unit structures have to be provided as algorithm input in the form of SMARTS, which requires a priori knowledge of the identity of repeating units and familiarity with SMARTS syntax. On one hand, this requirement makes OngLai highly suited to its original intended application, which is to aid in the identification of unknown but related features in mass spectra. In this case, repeating units are typically known from the outset, as their structures can be deduced from the constant *m/z* differences between HRMS features (e.g., *m/z* = 14.0157 difference between features likely indicates that the repeating unit is CH_2_). However, from a pure cheminformatics perspective, homologous series classification should ideally be achievable without prior knowledge of repeating unit identity. Developing and implementing such an approach poses a complex but relevant problem, which could be addressed using maximum common substructure (MCS) detection functionality [41, 42] in an all-versus-all approach. That said, the necessity to determine the MCS of every molecule with every other molecule in the given dataset is potentially problematic due to the exponential scaling of required computation that is exacerbated when dealing with large chemical structures like polymers or certain natural products. Common cheminformatics methods like pre-screening and filtering repeating unit-less molecules to overcome these time-consuming MCS functionalities could be explored. Alternatively, parallelisation would be applicable here because the MCS of one molecule pair can be determined separately from the other pairs.

Another idea to approach the problem of homologous series detection is to employ spherical substructures of molecules, also called atomic environments, as used in molecular signatures [76], Morgan fingerprints [77, 78], or HOSE codes [79]. The first step would be to generate spherical substructures of different heights for every atom in a molecule, where a substructure of height 0 contains only the centre atom itself, the substructure of height 1 contains the centre atom and its direct neighbours, etc. For each height, the number of unique spherical substructures can be tracked. If there is a repeating unit in the molecular structure, there should be a detectable minimum in the diversity of a molecule’s spherical substructures for the height equal to the size of the repeating unit. This approach would have the advantage that it is dataset-independent, unlike the current or MCS approach, but would require many specific rules or heuristics for corner cases and a very fine tuning of the parameters for the detection of the assumed height that matches the repeating unit size, if a generally applicable parameter set can be identified at all.

A less complex application of spherical substructure approaches might also be used to detect repeating unit chains with an a priori definition of the repeating substructure that is searched for, as in this work. Instead of SMARTS-based matching as used here, spherical substructures of a matching height for one molecule would be generated and matched with the pre-defined repeating units to identify homologous compounds by their chains. The set height of the included atom neighbours could then be gradually increased to include the neighbouring repeating units until the structure no longer fits the predefined repeating unit structure. This way, a repeating unit chain could be detected directly as a coherent substructure. A disadvantage of the approach would be that spherical substructure notations like HOSE codes are more complex to define by hand and provide less options than SMARTS definitions, since they were not originally developed for substructure matching.

Beyond the classical methods of structural cheminformatics, further alternative approaches could employ machine learning. For example, one could define the problem as a classification task by training a model to recognise homologous vs. non-homologous molecules based on their SMILES strings or even structure depictions. In both data structures, repetitive repeating unit patterns should be detectable in a straightforward manner. A more complex alternative would be to extract the core and (in a generalised model) repeating unit structures, e.g., as SMILES strings. Current successes in similar applications are encouraging [80] but available training data would be a limiting factor, as the numbers of homologous structures detected in relevant datasets reported above and of published homologous series e.g., in specialised databases, appear too low for most machine learning tasks. However, defining core structures with chain attachment points and multiple repeating units structures may allow training data to be synthetically generated through recombination and enumeration to form diverse homologous series structures.

## Conclusions

OngLai is an open source algorithm implemented in RDKit that classifies homologous series within compound datasets based on two inputs: a CSV file containing compound SMILES representations and a repeating unit represented by a SMARTS string. Using the SMARTS definition of the repeating unit, OngLai first detects suitable cores by molecule fragmentation prior to series classification. Homologous series classification was demonstrated by applying OngLai to three open datasets: NORMAN-SLE, PubChemLite for Exposomics, and COCONUT. Thousands of homologous series with CH_2_ repeating units were detected within these datasets using the default algorithm settings. The results were validated using published homologous series and structure categories for surfactant and PFAS examples, and compared with the splitPFAS method for categorising PFAS. Both validation and comparison generally showed good agreement, with OngLai proving to be more granular in its detection of homologous series in some scenarios.

Overall, homologous series classification bears several advantages. Firstly, the detection of homologous series in datasets such as NORMAN-SLE and PubChemLite may support their identification using (LC-)HRMS. Homologous mass spectral features are frequently detected at high intensities in environmental samples and may form a large proportion of measured features that typically remain unknown (but are suspected to be compounds in chemical consumer products that are heavily produced and used, like surfactants). OngLai’s results could support the characterisation of these unknowns by providing researchers with classified homologous series within datasets, so they can perform more effective database matching of homologous features detected in their samples in a group-wise fashion. Such steps would contribute to tackling the problem of identifying and characterising UVCBs in the environment and further our understanding of the effects of chemical exposure and its impacts on the environment and/or disease, with the ultimate goal of protecting human health and the environment [26].

Secondly, the characterisation of chemical spaces is enhanced by identifying similar or related compounds that could be considered as a group. As OngLai essentially performs a type of clustering by grouping together homologous compounds, applying it to large screening datasets is a viable method for analysing large chemical spaces of interest and supporting the design of diverse molecule screening decks, which are of interest in drug discovery [70, 71]. An additional benefit accrued from chemical space characterisation via homologous series detection is that classified series can contribute to more efficient dataset curation, as mentioned with respect to polyfluorinated compounds found in the COCONUT dataset.

OngLai is freely and openly available on https://github.com/adelenelai/onglai-classify-homologues. Users are invited to apply OngLai on chemical datasets of interest, possibly as a first data exploration step, to uncover homologous compounds in their datasets, which may lead to insights on potential chemical groups, open new avenues for property prediction, and/or facilitate analytical detection. OngLai can also be used as a means for chemical grouping or to validate existing approaches, which may be of particular interest to e.g., regulatory stakeholders in environmental chemistry [81].

## Supplementary Information

**Additional file 1.** File containing links to code, datasets, and complete results described in the manuscript.

## Data Availability

OngLai homologue detection algorithm source code: Apache 2.0 Licence; https://github.com/adelenelai/onglai-classify-homologues. Software Requirements: Python 3.7 or higher, RDKit v2021.09.4 or higher, datamol v.0.7.3 or higher. Specific versions of the datasets used (NORMAN-SLE, PubChemLite for Exposomics, and COCONUT), as well as complete results, Python scripts and supporting files are freely available within the Supplementary Information archive on Zenodo: https://doi.org/10.5281/zenodo.7035020. The Additional file (.doc) contains details of the above archive.

## Abbreviations

COCONUT: COlleCtion of Open Natural ProdUcTs
HOSE: Hierarchically ordered spherical description of environment
LC-HRMS: Liquid chromatography-high resolution mass spectrometry
MCS: Maximum common substructure
NORMAN-SLE: NORMAN Suspect List Exchange
PFAS: Per- and Polyfluoroalkyl substances
PubChemLite: PubChemLite for Exposomics
SMILES: Simplified Molecular Input Line Entry System
SMARTS: SMILES ARbitrary Target Specification
UVCB: Substances of Unknown or Variable composition, Complex reaction products, or Biological materials
