# Supplementary material for: An algorithm to classify homologous series within compound datasets
Source: J Cheminform. 2022 Dec 13;14:85. doi: 10.1186/s13321-022-00663-y (PMC9746203; doi:10.1186/s13321-022-00663-y)
Supplement: Supplementary file 1 — Additional file 1. File containing links to code, datasets, and complete results described in the manuscript. [file 13321_2022_663_MOESM1_ESM.docx]

Additional information for:

‘An Algorithm to Classify Homologous Series Within Compound Datasets’

Adelene Lai*^1,2^, Jonas Schaub^2^, Christoph Steinbeck^2^, Emma L. Schymanski^1^

ORCIDs: AL: [0000-0002-2985-6473](https://orcid.org/0000-0002-2985-6473), JS: [0000-0003-1554-6666](http://orcid.org/0000-0003-1554-6666). CS: [0000-0001-6966-0814](https://orcid.org/0000-0001-6966-0814),

ELS: [0000-0001-6868-8145](http://orcid.org/0000-0001-6868-8145).

Emails: [adelene.lai@uni.lu](mailto:adelene.lai@uni.lu), [jonas.schaub@uni-jena.de](mailto:jonas.schaub@uni-jena.de), [christoph.steinbeck@uni-jena.de](mailto:christoph.steinbeck@uni-jena.de), [emma.schymanski@uni.lu](mailto:emma.schymanski@uni.lu)

^1^Luxembourg Centre for Systems Biomedicine (LCSB), University of Luxembourg, 6 avenue du Swing, 4367, Belvaux, Luxembourg.

^2^Institute for Inorganic and Analytical Chemistry, Friedrich-Schiller University, Lessing Strasse 8, 07743, Jena, Germany.

*Corresponding Author: Adelene Lai ([adelene.lai@uni.lu](mailto:adelene.lai@uni.lu))

Table of Contents

[Section 1 - OngLai Algorithm and Datasets Used 2](#_Toc112861352)

[1.1 Github repository and Zenodo release 2](#_Toc112861353)

[1.2 Datasets Used: NORMAN-SLE, PubChemLite, and COCONUT 2](#_Toc112861354)

[Section 2 - OngLai Results (Homologous Series Classified) 3](#_Toc112861355)

[Section 3 - Validation of Homologous Series Classified by OngLai 3](#_Toc112861356)

[3.1 Validation Datasets 3](#_Toc112861357)

[3.2 Validation Analysis 4](#_Toc112861358)

[3.3 Validation Results 4](#_Toc112861359)

[Section 4 - Comparison of OngLai with splitPFAS 5](#_Toc112861360)

[4.1 splitPFAS Dataset 5](#_Toc112861361)

[4.2 OngLai Results (CF_2_ Homologous Series Classified in splitPFAS Dataset) 5](#_Toc112861362)

[4.3 Analysis of Homologous Series Classified by OngLai in splitPFAS Dataset 5](#_Toc112861363)

##

## Section 1 - OngLai Algorithm and Datasets Used

### 1.1 Github repository and Zenodo release

OngLai GitHub project repository

- <https://github.com/adelenelai/onglai-classify-homologues>

OngLai release archived on Zenodo

- <https://github.com/adelenelai/onglai-classify-homologues/releases/tag/v.1.1.0>

###

### 1.2 Datasets Used: NORMAN-SLE, PubChemLite, and COCONUT

OngLai was applied to 3 open datasets from environmental chemistry, exposomics, and natural products: NORMAN-SLE,^1,2^ PubChemLite,^3,4^ and COCONUT^5,6^ respectively. The exact versions of these datasets used in the manuscript are archived on Zenodo: <https://doi.org/10.5281/zenodo.7035020>

**Additional note for preparing COCONUT dataset:**

COCONUT_DB_2021-11.smi was downloaded from<https://coconut.naturalproducts.net/download> on 2022-03-16 and converted to COCONUT_DB_2021-11.txt by running the following in a UNIX command line:

> tr -s '[:blank:]' ',' <COCONUT_DB_2021-11.smi >COCONUT_DB_2021-11.txt

Then, the following header was added to COCONUT_DB_2021-11.txt manually in a text editor: "SMILES, Name"

##

##

## Section 2 - OngLai Results (Homologous Series Classified)

OngLai was applied to NORMAN-SLE, PubChemLite, and COCONUT with a different repeating unit specified (CH_2_, CCO, and CF_2_) for each run of the algorithm*.* All of OngLai’s outputs are in the respective following folders, each folder representing all outputs per run of OngLai, available on Zenodo: <https://doi.org/10.5281/zenodo.7035020>

- *normansle_98116/*
  - *output_normansle_CH2_98116/*
  - *output_normansle_CCO_98116/*
  - *output_normansle_CF2_98116/*
- *pubchemlit3_392465/*
  - *output_pclite_CH2_392465/*
  - *output_pclite_CCO_392465/*
  - *output_pclite_CF2_392465/*
- *coconut_406919/*
  - *output_coconut_CH2_406919/*
  - *output_coconut_CCO_406919/*
  - *output_coconut_CF2_406919/*

## Section 3 - Validation of Homologous Series Classified by OngLai

### 3.1 Validation Datasets

The following datasets were used for validation of OngLai’s classification results of NORMAN-SLE, as described in the Methods and Results. The Zenodo links provided below point to the specific versions used in the manuscript unless otherwise specified. All files used are available in the Supplementary Information folder on Zenodo:<https://doi.org/10.5281/zenodo.7035020>

Published Homologous Series with CH_2_ and CCO repeating units

- Schymanski, E. S7 | EAWAGSURF | Eawag Surfactants Suspect List, 2014. <https://doi.org/10.5281/zenodo.3549934>
- Alygizakis, N. S23 | EIUBASURF | Surfactant Suspect List from EI and UBA, 2018. <https://doi.org/10.5281/zenodo.2648765>

Published Structure Categories with CF_2_ repeating units

- 109 CSV files (1 per Structure Category) were downloaded from the PubChem Classification Tree via the ID Exchange Service on 2022-08-08, merged using the provided awk script, and manually curated as specified in the README
  - *pfas-per-strucat-pubchem-2022-08-08/*
- The original source of the PFAS list:
  - PFAS Wang, Z. S25 | OECDPFAS | List of PFAS from the OECD, 2018. <https://doi.org/10.5281/zenodo.6349061>

### 3.2 Validation Analysis

Python code used to perform the validation is provided within 2 Jupyter Notebooks. Corresponding outputs generated within the Notebook files are listed in the Zenodo repository: <https://doi.org/10.5281/zenodo.7035020>

Notebook to prepare the validation datasets for manual inspection of series and output CSVs

- *Validate-homologous-series-classified.ipynb*
  - *compare_alg_cf2_s25.csv*
  - *compare_alg_s7_CCO.csv*
  - *compare_alg_s7_CH2.csv*
  - *compare_alg_s23_CCO.csv*
  - *compare_alg_s23_CH2.csv*

Notebook to analyse the OECD PFAS Structure Categories

- *validation-alg-s23-cf2-merged-oecdpfas-strucats.ipynb*

### 3.3 Validation Results

Validation was performed manually using the files generated in Section 3.2. Results are in the XLS file in the Zenodo repository: <https://doi.org/10.5281/zenodo.7035020>

- *validation-results-normansle-s7-s23-CH2-CCO.xlsx*

###

###

##

## Section 4 - Comparison of OngLai with splitPFAS

OngLai was compared to an existing tool for categorising PFAS compounds developed by Sha et al. called splitPFAS.^7^

### 4.1 splitPFAS Dataset

For the comparison, the splitPFAS dataset was downloaded and used as input for OngLai with CF_2_ specified as the repeating unit. All files described below are in the Zenodo repository: <https://doi.org/10.5281/zenodo.7035020>

The source of the original XLS file in the Supplementary Information of Sha et al.

- <https://www.rsc.org/suppdata/c9/em/c9em00321e/c9em00321e1.xlsx>

Three of the tabs in the above XLS file were saved as separate CSV files so they could be analysed using Python code (below). The CSV files are named according to the names of the spreadsheet tabs.

- *esi-splitpfas-results-perfluoroalkanoyl.csv*
- *esi-splitpfas-results-sulfonyl.csv*
- *esi-splitpfas-results-fluorotelomer.csv*

Python code within a Jupyter Notebook was used to merge these files to generate the splitPFAS dataset (*n*=770)

- *compile-splitpfas-inputs-analyse-series.ipynb*
- *merged_splitpfas-results_hs-classification.csv*

### 4.2 OngLai Results (CF_2_ Homologous Series Classified in splitPFAS Dataset)

OngLai was applied to the 770 PFAS compounds in the file *merged_splitpfas-results_hs-classification.csv.* All of OngLai’s outputs are in the following folder on the Zenodo repository <https://doi.org/10.5281/zenodo.7035020>:

- *output_splitpfas_770_CF2/*

### 4.3 Analysis of Homologous Series Classified by OngLai in splitPFAS Dataset

Python code was used to generate the splitPFAS dataset and analyse the homologous series classified by OngLai in the splitPFAS dataset which is provided in a Jupyter Notebook (the same as in Section 4.1), available on the Zenodo repository <https://doi.org/10.5281/zenodo.7035020>.

- *compile-splitpfas-inputs-analyse-series.ipynb*

**References**

(1) Mohammed Taha, H.; Aalizadeh, R.; Alygizakis, N.; Antignac, J.-P.; Arp, H. P. H.; Bade, R.; Baker, N.; Belova, L.; Bijlsma, L.; Bolton, E. E.; Brack, W.; Celma, A.; Chen, W.-L.; Cheng, T.; Chirsir, P.; Čirka, Ľ.; D’Agostino, L. A.; Djoumbou Feunang, Y.; Dulio, V.; Fischer, S.; Gago-Ferrero, P.; Galani, A.; Geueke, B.; Głowacka, N.; Glüge, J.; Groh, K.; Grosse, S.; Haglund, P.; Hakkinen, P. J.; Hale, S. E.; Hernandez, F.; Janssen, E. M.-L.; Jonkers, T.; Kiefer, K.; Kirchner, M.; Koschorreck, J.; Krauss, M.; Krier, J.; Lamoree, M. H.; Letzel, M.; Letzel, T.; Li, Q.; Little, J.; Liu, Y.; Lunderberg, D. M.; Martin, J. W.; McEachran, A. D.; McLean, J. A.; Meier, C.; Meijer, J.; Menger, F.; Merino, C.; Muncke, J.; Muschket, M.; Neumann, M.; Neveu, V.; Ng, K.; Oberacher, H.; O’Brien, J.; Oswald, P.; Oswaldova, M.; Picache, J. A.; Postigo, C.; Ramirez, N.; Reemtsma, T.; Renaud, J.; Rostkowski, P.; Rüdel, H.; Salek, R. M.; Samanipour, S.; Scheringer, M.; Schliebner, I.; Schulz, W.; Schulze, T.; Sengl, M.; Shoemaker, B. A.; Sims, K.; Singer, H.; Singh, R. R.; Sumarah, M.; Thiessen, P. A.; Thomas, K. V.; Torres, S.; Trier, X.; van Wezel, A. P.; Vermeulen, R. C. H.; Vlaanderen, J. J.; von der Ohe, P. C.; Wang, Z.; Williams, A. J.; Willighagen, E. L.; Wishart, D. S.; Zhang, J.; Thomaidis, N. S.; Hollender, J.; Slobodnik, J.; Schymanski, E. L. The NORMAN Suspect List Exchange (NORMAN-SLE): Facilitating European and Worldwide Collaboration on Suspect Screening in High Resolution Mass Spectrometry. *Environ. Sci. Eur.* **2022**, *34* (1), 104. https://doi.org/10.1186/s12302-022-00680-6.

(2) NORMAN Network. *PubChem Classification Browser - NORMAN Suspect List Exchange Tree*. https://pubchem.ncbi.nlm.nih.gov/classification/#hid=101 (accessed 2022-04-04).

(3) Schymanski, E. L.; Kondić, T.; Neumann, S.; Thiessen, P. A.; Zhang, J.; Bolton, E. E. Empowering Large Chemical Knowledge Bases for Exposomics: PubChemLite Meets MetFrag. *J. Cheminformatics* **2021**, *13* (1), 19. https://doi.org/10.1186/s13321-021-00489-0.

(4) Bolton, E.; Schymanski, E.; Kondic, T.; Thiessen, P.; Zhang, J. (Jeff). PubChemLite for Exposomics, 2022. https://doi.org/10.5281/zenodo.6383860.

(5) Sorokina, M.; Merseburger, P.; Rajan, K.; Yirik, M. A.; Steinbeck, C. COCONUT Online: Collection of Open Natural Products Database. *J. Cheminformatics* **2021**, *13* (1), 2. https://doi.org/10.1186/s13321-020-00478-9.

(6) *COCONUT: Natural Products Online*. https://coconut.naturalproducts.net/download (accessed 2022-04-04).

(7) Sha, B.; L. Schymanski, E.; Ruttkies, C.; T. Cousins, I.; Wang, Z. Exploring Open Cheminformatics Approaches for Categorizing Per- and Polyfluoroalkyl Substances (PFASs). *Environ. Sci. Process. Impacts* **2019**, *21* (11), 1835–1851. https://doi.org/10.1039/C9EM00321E.
